# Unveiling Pharmacological Promise of *Mangifera indica* (Haribhanga) Peel Extract: Exploring an Untapped Cultivar Through Biochemical and Computational Approaches

**DOI:** 10.1155/sci5/6516268

**Published:** 2025-03-10

**Authors:** Mst. Hajera Khatun, Saad Ahmed Sami, Farhana Sultana Mim, Pappu Kumar, Ariful Islam, Injamam Al Mahamud Rian, Md. Ashikur Rahman, Sharmin Islam Riya, Md. Lokman, Al Mamun, Md. Anwarul Haque, Mst. Sarmina Yeasmin, G. M. Masud Rana, Jaytirmoy Barmon

**Affiliations:** ^1^Department of Pharmacy, School of Science and Technology, Varendra University, Rajshahi 6204, Bangladesh; ^2^Department of Pharmacy, Faculty of Biological Sciences, University of Chittagong, Chattogram 4331, Bangladesh; ^3^Department of Pharmacy, Faculty of Science, University of Rajshahi, Rajshahi 6205, Bangladesh; ^4^BCSIR Rajshahi Laboratories, Bangladesh Council of Scientific and Industrial Research, Rajshahi 6206, Bangladesh

**Keywords:** antiarthritic, antidiabetic, antimicrobial, antioxidant, GC–MS, *Mangifera indica*, molecular docking

## Abstract

The Haribhanga is one of the most renowned varieties of mango native to the Rangpur region of Bangladesh. The study aimed to explore the in vitro and in vivo pharmacological potentialities of the methanolic extract of *Mangifera indica* (Haribhanga) (MEMI) peel. The antioxidant, antimicrobial, and antiarthritic activities of MEMI peel were conducted by the 2,2-diphenyl-1-picrylhydrazyl (DPPH) free radical scavenging, disc diffusion, and protein denaturation assays, respectively. The extract was administered to STZ-induced diabetic mice for 7 days for the observation of blood glucose, body weight, lipid profile, and liver enzyme levels. The gas chromatography–mass spectrometry (GC–MS) analysis was performed to identify phytochemicals in the extract. Subsequently, molecular docking was conducted to predict the binding affinity of the identified compounds. The MEMI peel exhibited notable antioxidant potentiality with an IC_50_ value of 4.43 ± 0.68 μg/mL and antimicrobial activity against *Bacillus cereus* with a zone of inhibition of 20.67 ± 1.52 mm. Furthermore, MEMI peel demonstrated substantial antiarthritic activity, with the highest inhibition of denaturation of protein (88%) observed at the highest dose (500 μg/mL). In the in vivo experiments, MEMI peel led to a significant increase in high-density lipoprotein (*p* < 0.001, *p* < 0.05), with a significant decrease in blood glucose (*p* < 0.001), triglycerides, total cholesterol, and low-density lipoprotein (*p* < 0.0001) in STZ-induced diabetic mice. Comparing the diabetic control mice, the MEMI peel substantially decreased (*p* < 0.001) the high serum levels of aspartate aminotransferase and alanine aminotransferase. Moreover, the extract significantly improved the body weight (*p* < 0.001) of diabetic mice after 7 days of treatment. GC-MS analysis identified 28 bioactive compounds, primarily fatty acid esters in the MEMI peel. Di-n-octyl phthalate, terpinen-4-ol, 8,11,14-docosatrienoic acid methyl ester, and phenol, 2-methoxy-4-(2-propenyl)-acetate exhibited the most favorable binding potential in molecular docking studies. The results suggest that MEMI peel possesses antimicrobial, antiarthritic, antidiabetic, antihyperlipidemic, and liver enzyme protective activities as a promising antioxidant.

**Summary**

• Methanolic extract of *Mangifera indica* (MEMI) peel demonstrated significant antioxidant, antiarthritic, and antimicrobial properties.
• MEMI peel exhibited prominent antidiabetic, antihyperlipidemic, and liver enzyme protective activity.
• Gas chromatography–mass spectrometry (GC–MS) analysis revealed the presence of 28 bioactive compounds in MEMI peel.
• Bioactive compounds of MEMI peel supported the observed pharmacological activities in the molecular docking study.

## 1. Introduction

Diabetes mellitus, characterized by persistent hyperglycemia, presents a significant global health burden, affecting millions worldwide with projections indicating a rising trend [1–4]. Among its various forms, Type 1 and Type 2 diabetes mellitus are predominant [5]. Type 1 diabetes results from insulin deficiency, necessitating insulin therapy, while Type 2 diabetes involves insulin resistance leading to fasting hyperglycemia [6]. Both types entail prolonged health complications affecting multiple organs [7, 8] and increased susceptibility to bacterial infections [9], particularly in rheumatoid arthritis patients who also exhibit a heightened risk of developing Type 2 diabetes [10, 11]. Oxidative stress, resulting from hyperglycemia-induced reactive oxygen species, is implicated in the pathogenesis of diabetes and its complications, highlighting the importance of antioxidant interventions in disease management [11–13].

Current diabetes treatments predominantly rely on pharmaceutical agents, yet achieving optimal glycemic control remains a challenge for many patients, necessitating the exploration of novel therapeutic avenues [14–16]. Traditional plant remedies offer promising alternatives due to their efficacy, safety, and minimal side effects [17].

The “Haribhanga” mango (also known as Harivanga and scientific name *Mangifera indica* L.) is a mango cultivar grown in Bangladesh's northwest, particularly in the Rangpur area [18]. Abdus Salam Sarkar first introduced Haribhanga in the year 2003 [18]. The large-scale cultivation of it is currently taking place in the Rangpur district as well as other parts of the northern region [19]. These mangoes are spherical and black in appearance and usually weigh between 200 and 400 g and are extremely meaty and fiberless. They have been found to weigh as much as 700 g. *M. indica* has been identified for various pharmacological activities like antidiabetic, antihyperlipidemic, hepatoprotective, antioxidant, anticancer, anti-inflammatory, antidiarrheal, and antimicrobial activities [20, 21]. Moreover, mango peels are a great source of phytochemicals such as carotenoids [22, 23], phenolic compounds [22, 24, 25], and flavonoids [26] as well as also contain energy, dietary fiber, carbs, protein, and lipids [24, 27, 28]. These bioactive chemicals are of great interest because of their strong antioxidant properties and potential medicinal benefits [29–33]. However, a number of variables, including variety, maturation stage, soil type, production location, farming techniques, and processing circumstances, affect the amount and quality of bioactive chemicals in mango peel [34]. Characterization is a crucial stage in making these by-products from mangoes valuable, whether they are used in the food business or other industries [35]. Therefore, the Haribhanga mango, a cultivar native to Bangladesh, has garnered attention for its potential medicinal properties, particularly its peel, which remains largely unexplored scientifically. Furthermore, by assessing exploratory and safety investigations, computational methods have provided a groundbreaking framework for identifying innovative treatment regimens, paving the way for a more rapid, cost-effective, and productive transition into the healthcare facility. In this insight, molecular docking is a useful tool to predict the pharmacological potential of identified compounds from different plant extracts [36].

This study aimed to investigate the therapeutic potential of the methanolic extract of *Mangifera indica* L. (Haribhanga) peel (MEMI) in streptozotocin (STZ)-induced diabetic mice. Evaluation encompassed in vitro assessments of antimicrobial, antioxidant, and antiarthritic activities, alongside in vivo studies focusing on antidiabetic, liver enzyme protective, and antihyperlipidemic effects. Additionally, in silico molecular docking simulation was employed to predict the binding affinity of bioactive compounds identified in MEMI peel extract against various target receptors relevant to the investigated activities [36]. By integrating experimental and computational approaches, this research sought to delineate the pharmacological profile of MEMI peel extract, shedding light on its potential therapeutic applications in managing diabetes and associated complications, while also providing insights into its antimicrobial, antioxidant, and antiarthritic properties.

## 2. Materials and Methods

### 2.1. Collection of the Plant

*M. indica* L. (Haribhanga) fruits weighing 10 kg were collected in July 2019 (monsoon season in Bangladesh) from Dinajpur district for this study. The collected plant species were identified by the taxonomist Professor Dr. A. H. M. Mahbubur Rahman, Department of Botany, University of Rajshahi, Bangladesh (Voucher No. RR665).

### 2.2. Preparation of the Extract

The collected mangoes were used after they had completed ripening. Samples of peels were separated manually from mango fruits, washed carefully with tap water, and rinsed with distilled water. A total of 1505 g of fresh peels were obtained from 10 kg of mango. The peels were then sundried for 1 week for complete drying. Using the proper grinder, they were ground into a coarse powder resulting in 776 g (51.56%) of weight. The extract was prepared by the previously described solvent system which involved an extraction method with a slight modification [37]. A clean glass jar with a flat bottom is used to contain about 200 g of powdered sample and is immersed in 500 mL of methanol. The glass jar along its contents were sealed and stored for 7 days with sporadic stirring and shaking. The whole mixture was then coarsely filtered by a piece of clean and white cotton material. Afterward, it was again filtered using Whatman filter paper (Bibby RE200, Sterilin Ltd., UK). Using a vacuum rotary evaporator, the filtrate was concentrated at 50°C. It produced a reddish-black, sticky concentrate extract weighing around 15 g (percentage yield: 7.5%). For future usage, it was stored in a sealed glass vial and kept in a refrigerator (at 4°C temperature).

### 2.3. Phytochemical Screening

The main secondary metabolites such as phenolic compounds, saponins alkaloids, cardiac glycosides, tannins, flavonoids, and terpenoids were detected using standard procedures [38–40].

### 2.4. GC–MS Analysis

MEMI peel used for GC–MS analysis. In order to prepare the sample, 2 mL of GC-grade methanol (Sigma-Aldrich) was added to 20 mg of the extract. The resultant mixture was effectively mixed with a vortex mixer and then filtered using a syringe filter. The prepared sample was subsequently transferred into a GC vial, and 4 μL of the solution was injected into the GC–MS machine (SHIMADZU GC-MS QP-2020) using helium as a carrier gas with a flow rate of 1.7 mL/min and flow pressure of 115.8 kPa. The oven's temperature was set to start at 80.0°C and hold for 2.0 min, then rise 7°C/min to 150.0°C (hold for 5.0 min), and then to 280.0°C (hold for 5.0 min). The ionization mass spectroscopic analysis was performed at 70 eV, and mass spectra were recorded in the range of 50 m/z to 500 m/z for a duration of 40.56 min. The cutoff time for the solvent was 3 min. By matching the mass spectra to those in the NIST08s, NIST08, and NIST14 libraries, components were identified.

### 2.5. Antioxidant Activity Test

The standard approach was used to dictate the antioxidant capacity of the extract through the 2,2-diphenyl-1-picrylhydrazyl (DPPH) stable free radical scavenging activity [41]. In detail, 3.0 mL of the methanol solution of DPPH was mixed with various concentrations of the extract. Then, the antioxidant capacity was measured by the ability of the extract to bleach a purple-colored DPPH methanol solution by determining the absorbance using a UV spectrophotometer (UV–1501PC SHIMADZU, Japan) at 517 nm. As a positive control, butylated hydroxytoluene (BHT) was used. Using the following equation, the percentage of the DPPH free radical scavenging was calculated:

(1) $$\begin{array}{c} \%\text{ of scavenging of DPPH radical}=\left[\frac{A_{1}-A_{2}}{A_{1}}\;\right]\times 100, \end{array}$$

where *A*_1_ = absorbance of control and *A*_2_ = absorbance of sample solution.

Then, % of DPPH scavenging was plotted against the different concentrations utilized, and the IC_50_ (the concentration of the sample required to scavenge 50% of DPPH) was determined from the straight line (linear regression) using the equation *y* = *mx* + *c*, where IC_50_ or *x* = (50 − *c*)/*m* by MS Excel 2013 software. The higher scavenging power of the radical by the extract is implied by a lower IC_50_ and vice versa.

### 2.6. Antimicrobial Activity Test

The antimicrobial efficacy of the MEMI peel was assessed using the disc diffusion technique [42]. Using a micropipette, dried and sterile filter paper discs (5 mm in diameter) were impregnated with 10 μL of the diluted extract solution (50 mg/mL) in an aseptic hood, which was then allowed to dry out. Then, discs containing the extract (500 μg/disc) were dispensed onto the surface of the inoculated agar plate with the pathogenic test microorganisms (1 × 10^6^ cfu/mL). Blank discs (impregnated with solvents) and standard kanamycin discs (30 μg/disc) were regarded as negative and positive controls, respectively. Then, the agar plates were maintained at 4°C for an hour in order to diffuse the test substance. Therefore, the plates were incubated for 24 h at 37°C to promote microbial growth. A clear, definite zone of inhibition observed surrounding the discs having the ability of the test material to impede the growth of microorganisms. Each sample was examined in triplicate, and the zones of inhibition in mm (including the 5-mm disc) were determined.

### 2.7. Antiarthritic Activity Test

The albumin denaturation method [43] was used to assess this activity with minor modifications. Reaction mixture of each concentration of MEMI peel (100, 250, and 500 μg/mL) was prepared by mixing 100 μL of the test sample and 100 μL of bovine serum albumin (BSA) (5%) solution, and then, pH was adjusted to 6.3 by adding a small amount of 1 N HCl. These solutions were incubated for 20 min at 37 ± 1°C. Therefore, the extract solutions were kept for 10 min in a water bath at 70°C in order to cause denaturation. After cooling the solutions, turbidity was determined at 660 nm by spectrophotometer (UV–1501PC SHIMADZU, Japan). Diclofenac sodium was utilized as the reference standard and handled identically as test extracts. Each experiment was performed in triplicate, and the average was calculated. The following equation was used to determine the % inhibition of protein denaturation:

(2) $$\begin{array}{c} \%\text{ inhibition of protein denaturation}=\left[\frac{A_{1}-A_{2}}{A_{1}}\right]\times 100, \end{array}$$

where *A*_1_ = absorbance of control and *A*_2_ = absorbance of test sample.

### 2.8. Experimental Animals

White Swiss albino mice (female) that were 35–37 days old and weighed between 28 and 33 g were used in the investigation. The mice were maintained in a controlled environment (temperature: 23.0 ± 2.0°C, relative humidity: 55%–65% and 12 h light-dark cycle) before starting the experiment and were provided standard feed and water *ad libitum*. Prior to the studies, the animals spent a month becoming adapted to the laboratory environment. All study protocols for animal experiments were approved by the animal morals guidelines of the institutional “Ethical Review Committee” of Varendra University, Rajshahi, Bangladesh (VU/ERC/2021-2022/004).

### 2.9. Acute Toxicity Test

The mice were separated into eight groups (five mice per group). The first seven groups (12-h fasted mice) received varying doses of the extract (25, 50, 100, 250, 500, 1000, and 2000 mg/kg body weight p.o., respectively), whereas the normal control mice received only the vehicle. The autonomic profiles, neurological changes, and behaviors of all treated mice were all recorded every 2 h for 24 h [44]. Then, according to the guidelines of the Organization for Economic Cooperation and Development (OECD) Guideline, 2008, the animals were followed for another two weeks to determine the mortality rate [45].

### 2.10. Induction of Diabetes

After a month of acclimation into the laboratory environment, a single intraperitoneal injection of STZ (45 mg/kg) prepared in a 0.01M citrate buffer (pH of 4.5) was given to develop diabetes mellitus [46]. To prevent hypoglycemic mortality, 5% w/v of sucrose solution was administered orally to the mice for 24 h of STZ injection. The control mice received intraperitoneal injections of citrate buffer. Three days after receiving the STZ injection, a few drops of blood were drawn from a tail vein and used to measure the glucose levels with a glucometer (Safe Touch Blood Glucose Meter, Germany). Mice were selected as diabetic for the investigation if their blood glucose levels were greater than 11 mmol/L [47–49]. The Type II diabetes was developed.

### 2.11. Experimental Design

The following five groups, each with four animals, were used for the study:

  Group 1: Normal control
  Group 2: Diabetic control
  Group 3: Standard (Linagliptin 5 mg/70 kg BW) in diabetic mice
  Group 4: MEMI (100 mg/kg BW) in diabetic mice
  Group 5: MEMI (200 mg/kg BW) in diabetic mice

Tween 80 (1% w/v in water) at a dose of 10 mL/kg was given orally to the animals in Groups 1 and 2. Linagliptin (5 mg/kg BW) and MEMI peel (100 mg/kg and 200 mg/kg BW) were administered to Group 3, Group 4, and Group 5, respectively. Intragastric feeding tubes were used to provide the standard drug, the extracts, and 1% Tween 80 in water to mice for 7 days. Body weight fluctuations were tracked on the first day of the trial, the third day, and the seventh day of all the experimental mice. A few drops of blood were drawn from the vein of the tail at days 0, 3, and 7 to measure blood glucose levels by glucometer. All the overnight starved mice were anesthetized with 1.7% isoflurane using the drop method [50] after completion of the experimental period, and the thoracic artery was then opened after cutting the abdomen skin and blood was taken directly from the heart's aorta by heparinized syringes. The serum utilized for the biochemical study was obtained from the centrifugation of the blood for 30 min at 4000 rpm.

### 2.12. Biochemical Analysis

Using serum, the biochemical parameters like triglycerides (TG), total cholesterol (TC), high-density lipoprotein (HDL), low-density lipoprotein (LDL), aspartate aminotransferase (AST), and alanine aminotransferase (ALT) were determined by commercially available animal spectrophotometric assay kits (Human, Germany) in accordance with the manufacturer's instructions and absorbance was measured by UV–Vis spectrophotometer (UV–1501PC SHIMADZU, Japan).

### 2.13. Statistical Analysis

The mean ± standard deviation (SD) was determined for each group by Microsoft Excel 2013. The statistical differences among the groups were compared using a two-way analysis of variance (ANOVA) followed by a Bonferroni posttest using GraphPad Prism (Version 8.1). At this point, *p* < 0.05 was regarded as significant.

### 2.14. Molecular Docking Analysis

Molecular docking analysis was conducted based on the methodologies of Mowla et al. with minor modifications [51].

#### 2.14.1. Ligand Retrieval and Preparation

All 28 compounds identified from the MEMI peel through GC–MS analysis were chosen to undergo molecular docking. The compounds' 3-D conformers were downloaded in SDF format from the PubChem database [52]. After that, ligands were developed by reducing their energy form and converting them into PDBQT format from SDF format via a PyRx 0.8 module called Open Babel [53].

#### 2.14.2. Protein Preparation

From the PDB database, the 3-D crystal structure of the human erythrocyte catalase (PDB ID: 1DGH, resolution: 2.00 Å) [54], *Staphylococcus aureus* Gyrase B enzyme (PDB ID: 4URM, resolution: 2.94 Å) [55], BSA (PDB ID: 4OR0, resolution: 2.58 Å) [56], human DPP4 receptor (PDB ID: 2OQV, resolution: 2.80 Å) [57], human squalene synthase (PDB ID: 1EZF, resolution: 2.15 Å) [58], and tumor necrosis factor-alpha (TNF-alpha) converting enzyme (PDB ID: 2FV5, resolution: 2.10 Å) [59] were retrieved for predicting antioxidant, antibacterial, antiarthritic, antidiabetic, antihyperlipidemic, and hepatoprotective activities, respectively. At first, in the Discovery Studio (DS) version 4.5, all the heteroatoms and the water molecules from the protein structure were removed. With the utilization of GROMOS 9643B1 parameters, minimizations were executed in vacuo in the Swiss-PDB Viewer software [60]. Lastly, the structures were saved for further investigations in the BIOVIA DS 2019 in .pdb format [61].

#### 2.14.3. Molecular Docking

The active site residues of the chosen protein structures were predicted by exploiting the PDBsum database [62]. Molecular docking was performed in AutoDock Vina software using default parameters where AutoDock Vina was administered through the shell script by AutoDock Vina developers. Here, the grid box's size in relation to the *X*, *Y*, and *Z* axes was determined at 72.80 × 86.44 × 73.35 Å for the human erythrocyte catalase, 42.61 × 40.98 × 44.72 Å for the *Staphylococcus aureus* Gyrase B enzyme, 92.35 × 61.89 × 73.50 Å for the BSA, 75.72 × 75.99 × 74.87 Å for the human DPP4 receptor, 54.53 × 54.16 × 50.93 Å for the human squalene synthase, and 56.35 × 42.98 × 45.29 Å for the TNF-alpha converting enzyme, respectively. The cocrystallized ligand of each protein was considered as a control compound for both activities. Results of docking analysis are represented as docking scores in kcal/mol where the more binding affinity is determined by the greater negative scores. The binding affinity scores and amino acid interactions were compared with the control [63].

## 3. Results

### 3.1. Phytochemical Screening

Saponins, cardiac glycosides, tannins, flavonoids, terpenoids, cardiac glycosides, and phenolic compounds were found in the MEMI peel by phytochemical analysis; however, alkaloids were found to be absent (Supporting Table S1).

### 3.2. GC–MS Analysis

**Figure 1:**
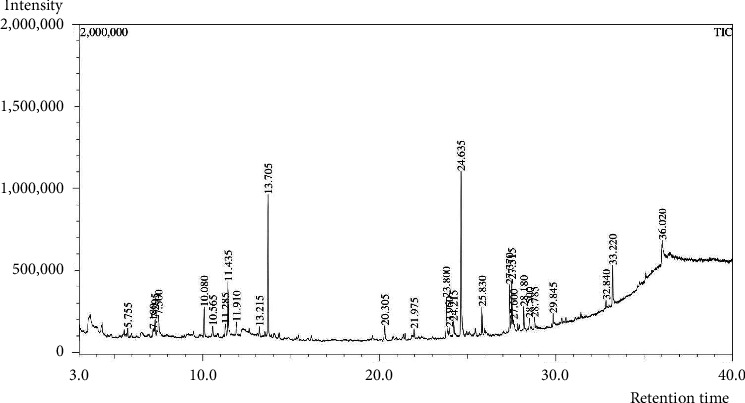
The total ionic chromatogram (TIC) of MEMI peel by GC-MS.

The MEMI peel contained a total of 28 compounds which were eluted between 3.0 and 40.0 min. Most of the compounds are hydrocarbons, phenolics, and fatty acid esters. The predominant compounds are found in hexadecanoic acid methyl ester (24.24%), and phenol, 3,5-bis(1,1-dimethylethyl) (21.06%). The description of the identified compounds is presented in Figure 1 and Table 1.

### 3.3. Antioxidant Activity Test

**Figure 2:**
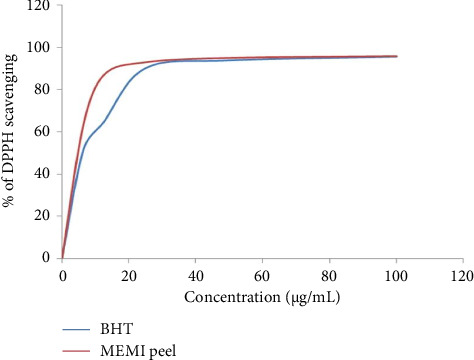
Antioxidant activity of MEMI peel (% of DPPH scavenging of MEMI peel and standard BHT).

The % of DPPH scavenging of the MEMI peel and the standard BHT has been shown in Figure 2. The IC_50_ of the MEMI peel and the standard BHT in the DPPH assay was 4.43 ± 0.68 µg/mL and 6.53 ± 0.53 μg/mL, respectively, as indicated in Figure 2 and Table 2.

### 3.4. Antibacterial Activity Test

The current research revealed that the MEMI peel exhibited various levels of antibacterial potential against all of the examined pathogens (Table 3). The highest zone of inhibition (20.67 mm) was found against *Bacillus cereus* followed by *Pseudomonas aeruginosa* (15.67 mm), *Staphylococcus aureus* (15.33 mm), and *Escherichia coli* (13.33 mm).

### 3.5. Antiarthritic Activity Test

**Figure 3:**
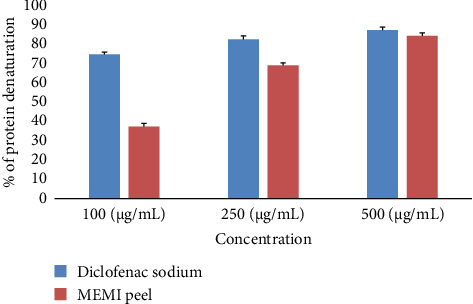
Antiarthritic activity of MEMI peel. The experiment was performed in triplicate (*n* = 3), and the % inhibition of protein denaturation of MEMI peel and standard diclofenac sodium was calculated from the average absorbance of three experiments.

The extract was found to be effective as an antiarthritic agent and exhibited activity in a concentration-dependent fashion (Figure 3). Hence, the maximum activity (88% inhibition of protein denaturation) observed at the highest dose (500 μg/mL), while 91% of protein denaturation was inhibited by the standard diclofenac sodium at the same dose.

### 3.6. Acute Toxicity Test

Based on the safety data gathered, the extract was deemed safe up to the dose of 2 g/kg BW. The absence of lethality, behavioral changes, or death during the course of the trial was meticulously observed in the animals (Supporting Table S2), and this is thought to have therapeutic benefits.

### 3.7. Effect of MEMI Peel on the Body Weight of STZ-Induced Diabetic Mice

**Figure 4:**
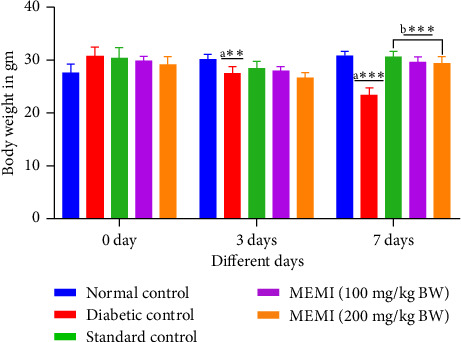
Effect of MEMI peel on the body weight of STZ-induced diabetic mice. Values are expressed as mean ± SD, *n* = 5 in each group. Data were analyzed by two-way ANOVA followed by Bonferroni posttest. a^∗∗∗^*p* < 0.001 and a^∗∗^*p* < 0.01, compared to normal control. b^∗∗∗^*p* < 0.001, compared to diabetic control.

The body weight of diabetic control mice (Group 2) was significantly lowered (*p* < 0.01 and *p* < 0.001) compared to that of the normal control mice (Group 1) at 3 and 7 days, respectively. Linagliptin (5 mg/70 kg) as well as the MEMI peel (100 and 200 mg/kg BW) treatment showed a slight decrease in body weight at day 3 but prevented this body weight reduction significantly (*p* < 0.001) than the diabetic control mice after completion of the treatment for 7 days (Figure 4).

### 3.8. Effect of MEMI Peel on Blood Glucose Level of STZ-Induced Diabetic Mice

**Figure 5:**
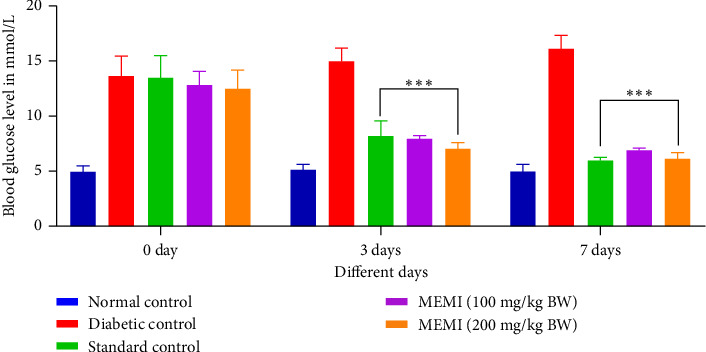
Effect of MEMI peel on the blood glucose level of STZ-induced diabetic mice. Values are expressed as mean ± SD, *n* = 5 in each group. Data were analyzed by two-way ANOVA followed by Bonferroni posttest. ⁣^∗∗∗^*p* < 0.001, compared to diabetic control.

The oral treatment by the MEMI peel (100 mg/kg and 200 mg/kg BW) in diabetic mice significantly lowered (*p* < 0.001) the blood sugar level to that of the diabetic control group both at the 3 days and after completion of the treatment for 7 days in a dose-dependent manner (Figure 5).

### 3.9. Effect of MEMI Peel on the Lipid Profile of STZ-Induced Diabetic Mice

**Figure 6:**
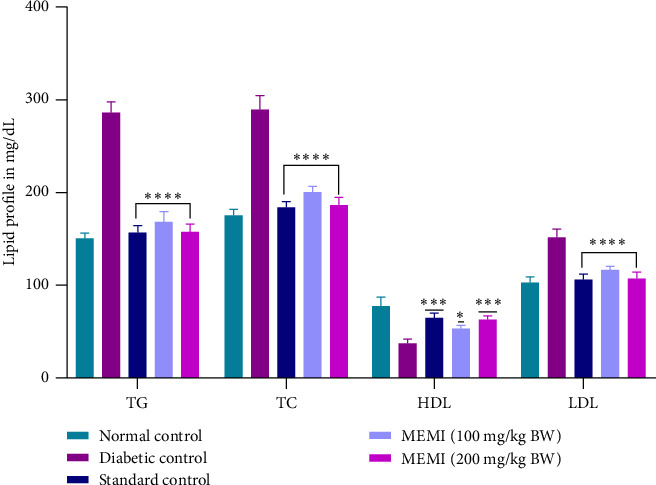
Effect of MEMI peel on the lipid profile of STZ-induced diabetic mice. Values are expressed as mean ± SD, *n* = 5 in each group. Data were analyzed by two-way ANOVA followed by Bonferroni posttest. ⁣^∗∗∗∗^*p* < 0.0001, ⁣^∗∗∗^*p* < 0.001, and ⁣^∗^*p* < 0.05, compared to diabetic control.

The administration of STZ severely altered the lipid profile of diabetic mice than that of the normal control mice. At the end of the experiment, the lipid profile was significantly (*p* < 0.0001, *p* < 0.001, and *p* < 0.05) decreased by the repeated daily treatment of MEMI peel (100 and 200 mg/kg BW) in diabetic mice for 7 days compared to the untreated mice (diabetic control group) (Figure 6).

### 3.10. Effect of MEMI Peel on the Liver Enzymes of STZ-Induced Diabetic Mice

**Figure 7:**
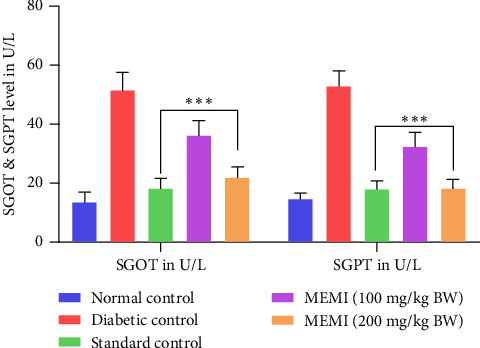
Effect of MEMI peel on the liver enzymes of STZ-induced diabetic mice. Values are expressed as mean ± SD, *n* = 5 in each group. Data were analyzed by two-way ANOVA followed by Bonferroni posttest. ⁣^∗∗∗^*p* < 0.001, compared to diabetic control.

The diabetic control mice exhibited a considerable increase in AST and ALT levels than the normal mice. The above indicators were significantly (*p* < 0.001) increased after repeated daily oral treatment of MEMI peel (100 mg/kg and 200 mg/kg BW) and standard linagliptin for 7 days when compared to the diabetic control group (Figure 7).

### 3.11. Molecular Docking Analysis

The active site residues of the targeted proteins were obtained from the PDBsum database. The active site for human erythrocyte catalase (PDB ID: 1DGH) included Pro151, His194, Phe198, Ser201, Arg203, Asn213, His235, Lys237, Val302, Trp303, Pro304, His305, Gln442, Phe446, and Val450 residues. The active site for *Staphylococcus aureus* Gyrase B enzyme (PDB ID: 4URM) included Asn54, Ser55, Glu58, Asp81, Arg84, Ile86, Gln91, Lys93, Met94, Ala98, Val101, Ile102, Leu106, His107, Ala108, Gly109, Gly110, Lys111, Thr173, and Ile175 residues. The active site for BSA (PDB ID: 4OR0) included Asn390, Phe402, Arg409, Tyr410, Lys413, Val432, Gly433, Cys437, Thr448, Leu452, Phe487, and Ser488 residues. The active site for the human DPP4 receptor (PDB ID: 2OQV) included Arg125, Glu205, Glu206, Val207, Ser209, Phe357, Arg358, Tyr547, Tyr631, Tyr662, Tyr666, Asn710, Val711, and His740 residues. The active site for human squalene synthase (PDB ID: 1EZF) included Arg52, Ser53, Phe54, Phe72, Tyr73, Leu76, Ala176, Gly180, Leu183, Gly208, Leu211, Gln212, Phe288, Pro292, and Met295 residues. The active site for TNF-alpha converting enzyme (PDB ID: 2FV5) included Gly346, Thr347, Leu348, Gly349, Leu350, Glu398, Leu401, His405, Glu406, His409, His415, Tyr433, Val434, Tyr436, Pro437, Ile438, Ala439, Val440, Ser441, Gly442, and Asn447 residues. The binding affinity scores of the identified compounds toward the targeted proteins are represented in Table 4.

#### 3.11.1. Molecular Docking Associated With Antioxidant Effects

**Figure 8:**
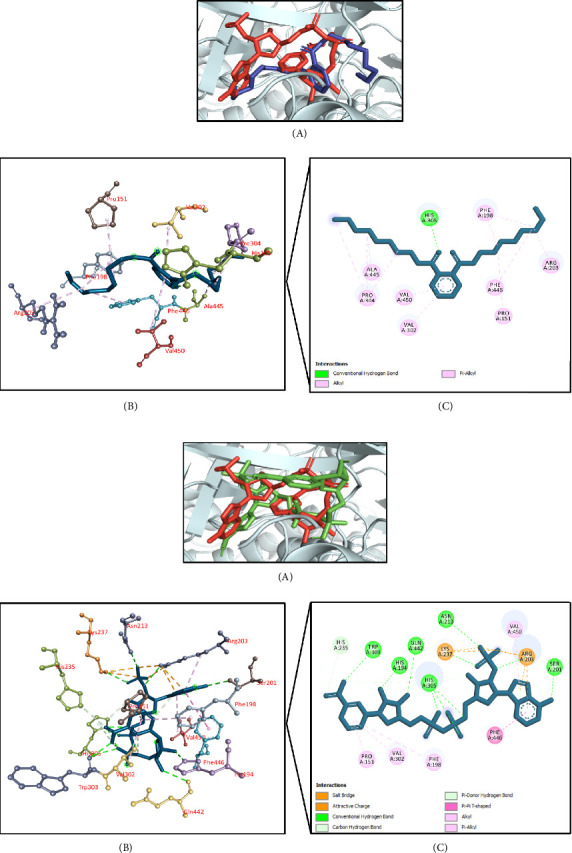
(a) Presentation of the best docking pose of di-n-octyl phthalate for antioxidant activity. (A) Superimposed illustration of di-n-octyl phthalate (blue stick) with the native cocrystallized ligand (red stick) at the active site of human erythrocyte catalase; (B) 3D illustration of interaction; and (C) 2D illustration of interaction. (b) Presentation of the best docking pose of control NADPH dihydro-nicotinamide-adenine-dinucleotide phosphate for antioxidant activity. (A) Superimposed illustration of docked cocrystallized ligand (green stick) with the native cocrystallized ligand (red stick) at the active site of human erythrocyte catalase; (B) 3D illustration of interaction; and (C) 2D illustration of interaction.

The results of molecular docking for antioxidant activity are summarized in Table 5. Using the protein known as human erythrocyte catalase (PDB ID: 1DGH) as a target receptor, this molecular docking simulation found that di-n-octyl phthalate had the highest docking interaction (−7.5 kcal/mol), followed by hexadecanoic acid, 1,1-dimethylethyl ester (−7.3 kcal/mol); 8,11,14-docosatrienoic acid methyl ester (−7.2 kcal/mol); 9-octadecenamide, (Z)- (−7.1 kcal/mol); and hexadecanoic acid, 2-methylpropyl ester (−7.0 kcal/mol) (Supporting Figure S1). The cocrystallized ligand, NADPH dihydro-nicotinamide-adenine-dinucleotide phosphate, had a docking score of −7.8 kcal/mol, and the score of di-n-octyl phthalate was close to that of the control. Among the docked compounds, di-n-octyl phthalate interacted with the active site of the targeted protein. It formed interactions with His305 residue through a conventional hydrogen bond, with Pro151, Phe198, Arg203, Val302, Pro304, Ala445, Phe446, and Val450 residues by hydrophobic (Pi-alkyl/alkyl) bonds (Figures 8(a), 8(b)).

#### 3.11.2. Molecular Docking Associated With Antibacterial Effects

**Figure 9:**
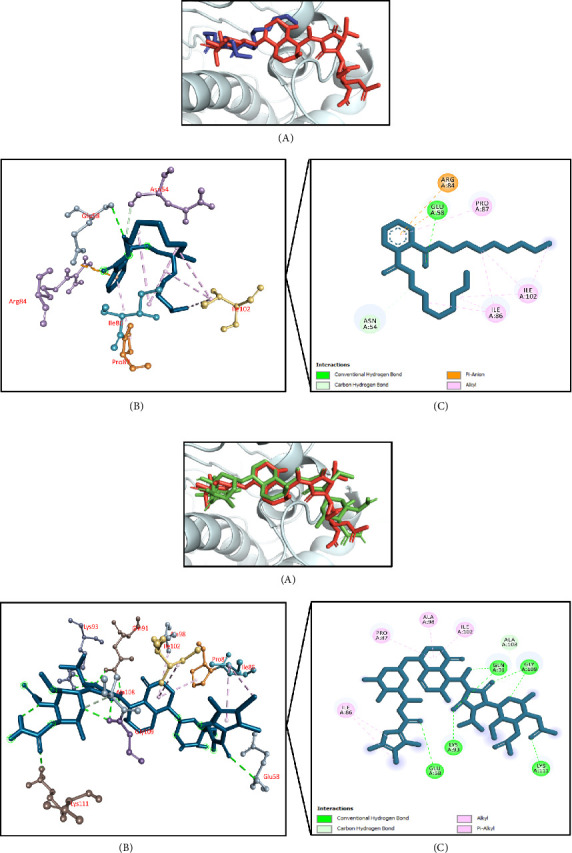
(a) Presentation of the best docking pose of di-n-octyl phthalate for antibacterial activity. (A) Superimposed illustration of di-n-octyl phthalate (blue stick) with the native cocrystallized ligand (red stick) at the active site of *Staphylococcus aureus* Gyrase B enzyme; (B) 3D illustration of interaction; and (C) 2D illustration of interaction. (b) Presentation of the best docking pose of the control XAM for antibacterial activity. (A) Superimposed illustration of docked cocrystallized ligand (green stick) with the native cocrystallized ligand (red stick) at the active site of *S. aureus* Gyrase B enzyme; (B) 3D illustration of interaction; and (C) 2D illustration of interaction.

Table 6 summarizes the antibacterial activity-related molecular docking simulation where the *Staphylococcus aureus* Gyrase B enzyme (PDB ID: 4URM) was used as a target receptor. It was found that di-n-octyl phthalate had the highest docking interaction (−6.5 kcal/mol), followed by caryophyllene (−6.4 kcal/mol); hexadecanoic acid, 2-methylpropyl ester (−6.1 kcal/mol); azulene (−6.0 kcal/mol); and phenol, 2-methoxy-4-(2-propenyl)-, acetate (−5.9 kcal/mol) (Supporting Figure S2). The cocrystallized ligand, XAM, had a docking score of −7.9 kcal/mol, and di-n-octyl phthalate bound mostly to the active site of the receptor among the docked compounds. It strongly interacted with the active site of the targeted protein by forming conventional hydrogen bonds with Glu58 residue, hydrophobic alkyl bonds with Ile86, Pro87, and Ile102 residues; a carbon-hydrogen bond with Asn54 residue; and an electrostatic pi-anion bond with Arg84 residue (Figures 9(a), 9(b)).

#### 3.11.3. Molecular Docking Associated With Antiarthritic Effects

**Figure 10:**
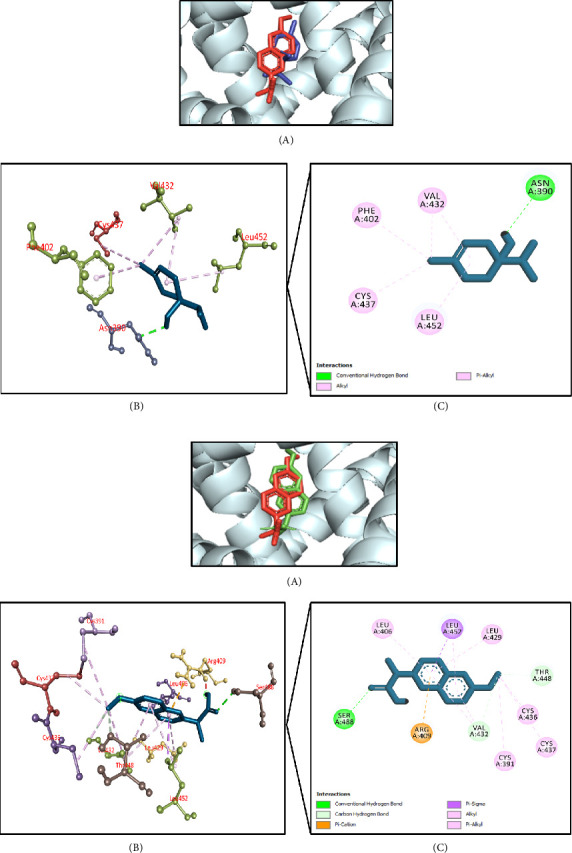
(a) Presentation of the best docking pose of terpinen-4-ol for antiarthritic activity. (A) Superimposed illustration of terpinen-4-ol (blue stick) with the native cocrystallized ligand (red stick) at the active site of bovine serum albumin; (B) 3D illustration of interaction; and (C) 2D illustration of interaction. (b) Presentation of the best docking pose of the control naproxen for antiarthritic activity. (A) Superimposed illustration of docked cocrystallized ligand (green stick) with the native cocrystallized ligand (red stick) at the active site of bovine serum albumin; (B) 3D illustration of interaction; and (C) 2D illustration of interaction.

Table 7 summarizes the antiarthritic activity-related molecular docking simulation where BSA (PDB ID: 4OR0) was used as a target receptor. It was found that terpinen-4-ol had the highest docking interaction (−7.6 kcal/mol) followed by tetradecane, 2,6,10-trimethyl- (−7.5 kcal/mol); phenol, 3,5-bis(1,1-dimethylethyl)- (−7.2 kcal/mol); caryophyllene (−7.0 kcal/mol); and methoxybicyclo[6.1.0]nona-2,4,6-triene (−6.9 kcal/mol) (Supporting Figure S3). The cocrystallized ligand, naproxen, had a docking score of −9.4 kcal/mol. Terpinen-4-ol strongly interacted with the active site of the targeted protein. It formed a conventional hydrogen bond with Asn390 residue, whereas hydrophobic (Pi-alkyl/alkyl) bonds were formed involving Phe402, Val432, Cys437, and Leu452 residues (Figures 10(a), 10(b)).

#### 3.11.4. Molecular Docking Associated With Antidiabetic Effects

**Figure 11:**
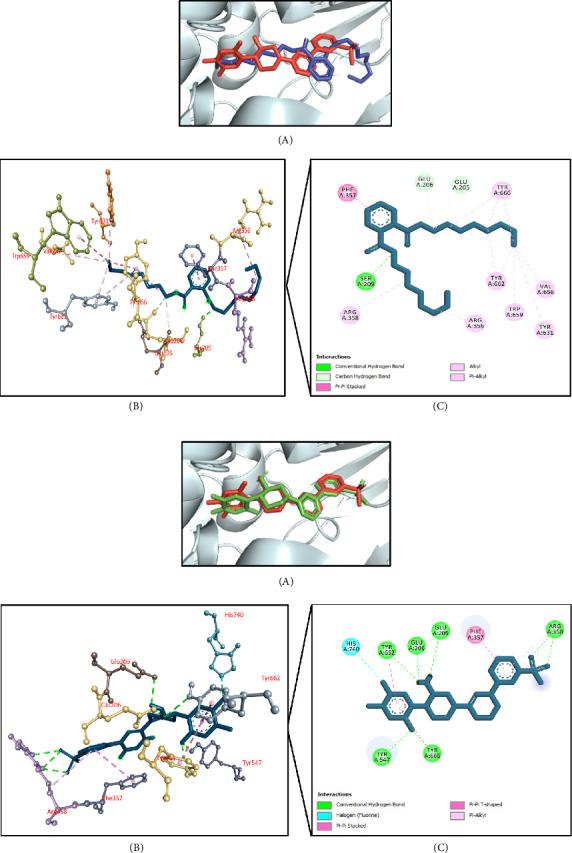
(a) Presentation of the best docking pose of di-n-octyl phthalate for antidiabetic activity. (A) Superimposed illustration of di-n-octyl phthalate (blue stick) with the native cocrystallized ligand (red stick) at the active site of human DPP4 receptor; (B) 3D illustration of interaction; and (C) 2D illustration of interaction. (b) Presentation of the best docking pose of control piperidine-constrained phenethylamine for antidiabetic activity. (A) Superimposed illustration of docked cocrystallized ligand (green stick) with the native cocrystallized ligand (red stick) at the active site of human DPP4 receptor; (B) 3D illustration of interaction; and (C) 2D illustration of interaction.

Table 8 summarizes the antidiabetic activity-related molecular docking simulation where human DPP4 receptor (PDB ID: 2OQV) was used as a target receptor. It was found that di-n-octyl phthalate and benzenemethanol, alpha-methyl-alpha-propyl-, demonstrated the highest docking interaction (−6.4 kcal/mol). They were followed by azulene (−6.3 kcal/mol), hexadecanoic acid, 2-methylpropyl ester (−6.3 kcal/mol), and phenol, 3,5-bis(1,1-dimethylethyl)- (−6.1 kcal/mol) (Supporting Figure S4). However, di-n-octyl phthalate had better interaction at the active site compared to the benzenemethanol, alpha-methyl-alpha-propyl-. The control piperidine-constrained phenethylamine had a docking score of −9.9 kcal/mol. Di-n-octyl phthalate demonstrated a strong interaction with the active site of the targeted protein. It formed a conventional hydrogen bond with Ser209 residue, hydrophobic bonds with Arg356, Phe357, Arg358, Tyr631, Val656, Trp659, Tyr662, Tyr666 residues, and carbon–hydrogen bonds with Glu205 and Glu206 residues (Figures 11(a), 11(b)).

#### 3.11.5. Molecular Docking Associated With Antihyperlipidemic Effects

**Figure 12:**
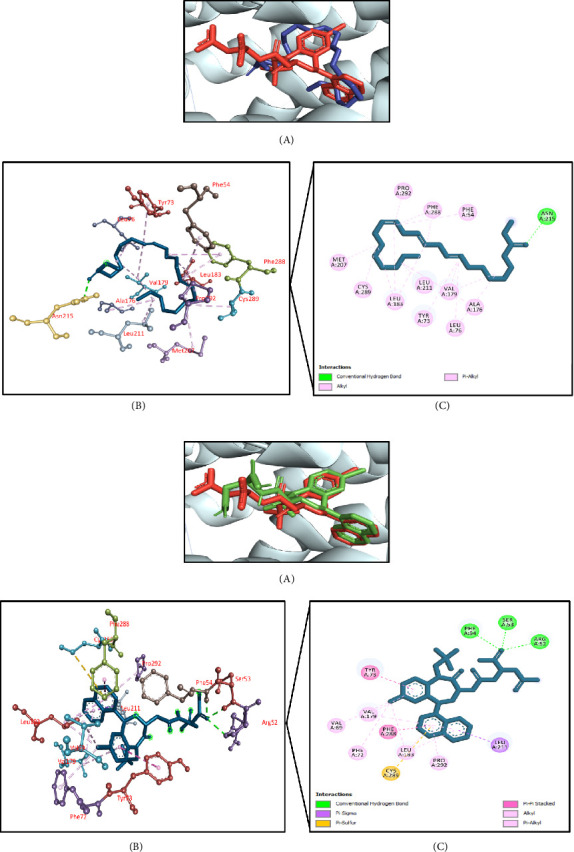
(a) Presentation of the best docking pose of 8,11,14-docosatrienoic acid methyl ester for antihyperlipidemic activity. (A) Superimposed illustration of 8,11,14-docosatrienoic acid, methyl ester (blue stick) with the native cocrystallized ligand (red stick) at the active site of human squalene synthase; (B) 3D illustration of interaction; and (C) 2D illustration of interaction. (b) Presentation of the best docking pose of control N-{2-[*trans*-7-chloro-1-(2,2-dimethyl-propyl)-5-naphthalen-1-yl-2-oxo-1,2,3,5-tetrahydro-benzo[e] [1, 4]oxazepin-3-yl]-acetyl}-aspartic acid for antihyperlipidemic activity. (A) Superimposed illustration of docked cocrystallized ligand (green stick) with the native cocrystallized ligand (red stick) at the active site of human squalene synthase; (B) 3D illustration of interaction; and (C) 2D illustration of interaction.

Table 9 summarizes the antihyperlipidemic activity-related molecular docking simulation where human squalene synthase (PDB ID: 1EZF) was used as a target receptor. It was found that 8,11,14-docosatrienoic acid methyl ester had the highest docking interaction (−7.6 kcal/mol) followed by hexadecanoic acid, 2-methylpropyl ester (−7.5 kcal/mol); caryophyllene (−7.5 kcal/mol); phenol, 3,5-bis(1,1-dimethylethyl)- (−7.1 kcal/mol); and di-n-octyl phthalate (−6.7 kcal/mol) (Supporting Figure S5). The docking score of the control N-{2-[*trans*-7-chloro-1-(2,2-dimethyl-propyl) -5-naphthalen-1-yl-2-oxo-1,2,3,5-tetrahydro-benzo[e] [1,4]oxazepin-3-yl]-acetyl}-aspartic acid was −11.6 kcal/mol. The top docked chemical 8,11,14-docosatrienoic acid methyl ester interacted with the active site of the targeted protein. It formed a conventional hydrogen bond with Asn215 residue and hydrophobic (Pi-alkyl/alkyl) bonds with Phe54, Tyr73, Leu76, Ala176, Val179, Leu183, Met207, Leu211, Phe288, Cys289, and Pro292 residues (Figures 12(a), 12(b)).

#### 3.11.6. Molecular Docking Associated With Hepatoprotective Effects

**Figure 13:**
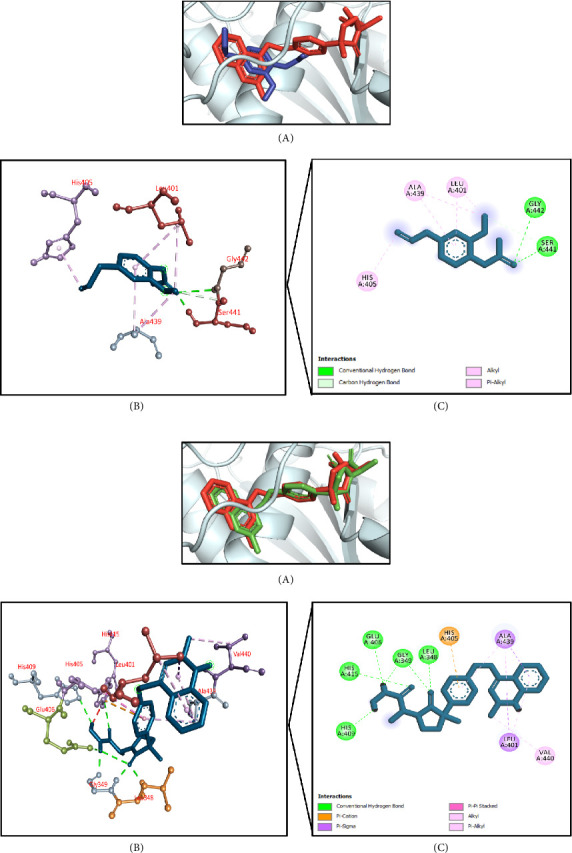
(a) Presentation of the best docking pose of phenol, 2-methoxy-4-(2-propenyl)-, acetate for hepatoprotective activity. (A) Superimposed illustration of phenol, 2-methoxy-4-(2-propenyl)-, acetate (blue stick) with the native cocrystallized ligand (red stick) at the active site of TNF-alpha converting enzyme; (B) 3D illustration of interaction; and (C) 2D illustration of interaction. (b) Presentation of the best docking pose of the control IK682 for hepatoprotective activity. (A) Superimposed illustration of docked cocrystallized ligand (green stick) with the native cocrystallized ligand (red stick) at the active site of TNF-alpha converting enzyme; (B) 3D illustration of interaction; and (C) 2D illustration of interaction.

The results of molecular docking for hepatoprotective activity are summarized in Table 10. Using TNF-alpha converting enzyme (PDB ID: 2FV5) as a target receptor, this molecular docking simulation found that phenol, 2-methoxy-4-(2-propenyl)-, acetate had the highest docking interaction at −7.8 kcal/mol. This was followed by terpinen-4-ol (−7.7 kcal/mol); phenol, 3,5-bis(1,1-dimethylethyl)- (−7.4 kcal/mol); benzenemethanol, alpha-methyl-alpha-propyl- (−7.0 kcal/mol); and di-n-octyl phthalate (−6.4 kcal/mol) (Supporting Figure S6). The docking score of the control IK682 was −11.1 kcal/mol. The top docked chemical phenol, 2-methoxy-4-(2-propenyl)-, acetate interacted with the active site of the targeted enzyme. It demonstrated two conventional hydrogen bonds with Ser441 and Gly442 residues, carbon–hydrogen bond with Ser441 residue, and hydrophobic (Pi-alkyl/alkyl) bonds with Leu401, His405, and Ala439 residues (Figures 13(a), 13(b)).

## 4. Discussion

Diabetes mellitus stands out as a significant global health concern in the 21^st^ century due to its prevalence and associated complications, such as chronic hyperglycemia and subsequent organ damage [64–66]. Management of diabetes focuses on controlling blood glucose levels to mitigate complications, yet available treatments often come with undesirable side effects. In this context, herbal medicines are gaining attention for their potential therapeutic benefits with fewer adverse effects [67–69]. Here, we investigated the potential pharmacological effects of the MEMI (Haribhanga) peel through in vitro, in vivo, and in silico assays.

We assessed MEMI peel for antimicrobial, antioxidant, and antiarthritic properties in vitro and its antidiabetic, antihyperlipidemic, and hepatoprotective effects in STZ-induced diabetic mice. In the pancreatic cells, STZ serves as a donor of nitric oxide and as a strong DNA methylating agent, and for low amounts of free radical scavenging enzymes, these cells are assailable to damage from nitric oxide and free radicals [70]. Linagliptin, a dipeptidyl peptidase-4 (DPP-4) enzyme inhibitor, is a great alternative when contemplating medications for people with Type 2 diabetes mellitus [71]. Our findings suggest that MEMI peel exhibits promising biological activities attributed to its phytochemical composition, including tannins, terpenoids, phenols, saponins, flavonoids, and glycosides [72]. GC-MS analysis revealed the presence of 28 phytochemicals in MEMI peel, predominantly fatty acid esters, which contribute to its therapeutic potential.

In vitro assays demonstrated MEMI peel's strong antioxidant activity, possibly due to the presence of phenolic compounds, flavonoids, and fatty acid esters, which scavenge free radicals [73–76]. MEMI peel also exhibited significant antimicrobial activity owing to the presence of the above-mentioned phytochemicals [77], particularly against *Bacillus cereus*, a common pathogen in diabetic patients [78, 79]. Xing et al. reported the antimicrobial activity against *Staphylococcus aureus* and *E. coli* using the aqueous extract of mango peel. The polyphenol in the extract was responsible for the activity [80]. Otherwise, a new cultivar (Haribhanga) of MEMI peel was demonstrated marked antibacterial activity against *Bacillus* cereus. Moreover, MEMI peel showed promising antiarthritic activity, attributed to its antioxidant potential and ability to inhibit protein denaturation [81].

In vivo studies in STZ-induced diabetic mice revealed that MEMI peel effectively lowered blood glucose levels, improved lipid profiles, and enhanced body weight compared to diabetic controls. Additionally, MEMI peel demonstrated hepatoprotective effects by reducing serum levels of liver enzymes AST and ALT, indicative of hepatocellular injury. Unrestrained free radicals created by protein glycosylation and glucose autooxidation due to hyperglycemia are crucial in the pathogenesis of diabetes [82]. According to previous research, the prominent antioxidant potential of MEMI peel that preserves pancreatic β cells could lead to restoring the function of β cells and contribute to reduce blood glucose levels in diabetic mice [68–70]. A significant improvement in the fatty acid mobilization from fat tissue and the unrestrained lipolytic hormonal effects on fat storage may both provide the development of marked hyperlipidemia in diabetes [75, 83]. Diabetes-related hyperlipidemia is distinguished by decreased HDL and increased TC, TG, and LDL levels. Patients with diabetes mellitus are more susceptible to coronary artery disease owing to the alterations of such parameters [84]. The chance of cardiovascular disease is inversely correlated with HDL and positively with LDL [74]. Furthermore, ROS-induced decreased insulin sensitivity is a crucial point in endothelial damage and cardiovascular problems associated with diabetes [85–88]. The presence of bioactive components and the extract's strong antioxidant activity may be linked to the reversal of the lipid profiles of the extract-treated mice [85, 86, 89]. Previous studies reported that peels of *M*. *indica* L. possessed antidiabetic and antioxidant potential and mainly phenolic compound exhibited the activity [20, 90, 91]. Mango peels of different varieties yielded varying amounts of bioactive chemicals, with larger concentrations of phenolic compounds showing greater antioxidant activity [91]. Furthermore, due to variations in the quantity and nature of their phytochemical contents, different *M. indica* cultivars displayed varying antioxidant potentials [92–94]. Nonetheless, in our study, MEMI peel of new cultivar pulled out phytochemicals mainly fatty acid esters found by GC-MS analysis and demonstrated more pronounced antidiabetic activity at lower doses of its unexplored cultivar. In addition, STZ-induced oxidative stress and hyperglycemia lead to liver dysfunction in diabetic mice [95, 96]. The MEMI peel restores liver function owing to the bioactive compounds and the prominent antioxidant potential of the extract [97, 98].

In order to support the biological activity of phytochemicals, it is critical to predict ligand–protein interactions. The biological action of plant extracts containing phytochemicals depends on their binding affinity and amino acid interactions with certain target proteins. To predict the interaction between a drug and a target protein's binding pocket, in silico molecular docking is commonly employed [99, 100]. For each activity in this study, we used this strategy to choose an established protein and depict its interactions and binding energy. Molecular docking studies were conducted on all 28 identified compounds isolated from the MEMI Haribhanga cultivar peel through GC–MS analysis. The computational method relies on the docking score generated by the interaction between the ligand and protein to predict activity. The stability of the complex is increased when the docking score decreases. To validate phytochemical biological activity, docking scores and interactions with target proteins are crucial [101].

Compounds were subjected to docking against human erythrocyte catalase for antioxidant activity. The catalase enzyme found in human red blood cells is essential for cellular defense against oxidative stress. Targeting catalase or developing molecules that improve its activity could be an effective approach in the process of drug design for disorders that are related to oxidative stress [54]. Di-n-octyl phthalate displayed the highest docking score against catalase and interacted with key Pro151, Phe198, Arg203, Val302, Pro304 Phe446, and Val450 residues through hydrophobic bonds and His305 by a strong conventional hydrogen bond. Di-n-octyl phthalate showed antioxidant potential in numerous experiments in previous studies [102]. All the phytochemicals were subjected to molecular docking against *Staphylococcus aureus* Gyrase B enzyme for antibacterial activity [103]. *Staphylococcus aureus* Gyrase B is an essential component of DNA replication and repair machinery. For *Staphylococcus* infections, blocking Gyrase B can be an effective approach in the context of bacterial illnesses. Antibiotics that target Gyrase B interfere with the replication of bacterial DNA, which in turn inhibits the growth of pathogens and helps to treat bacterial infections [104]. Di-n-octyl phthalate showed the strongest docking affinity against Gyrase B enzyme and interacted with key binding residue Glu58 through conventional hydrogen bond; with Ile86, Pro87, and Ile102 by hydrophobic bond; Asn54 through carbon–hydrogen bond; and Arg84 by electrostatic pi-anion bond, whereas the control did not bind with Asn54 and Arg84 residues which makes it evident that it is a valuable competitor to predict the antibacterial activity. Importantly, the phthalate derivatives exhibited pronounced antibacterial activity in previous studies [105]. Phytochemicals were docked against BSA for antiarthritic activity. BSA is frequently utilized as a template protein in a variety of investigations, including the development of new drugs. It is possible that researchers will use BSA in the context of arthritis in order to gain a better understanding of the interaction between drugs and proteins. It is possible for BSA to imitate certain characteristics of human serum albumin (HSA), which is the most abundant protein in human blood. The enzyme HSA has a role in the regulation of inflammation. It is possible that HSA plays a role in moderating the inflammatory processes that are associated with arthritis, which is characterized by an imbalance in the immune response. The function of HSA in the blood is to transport a wide variety of substances, including drugs. Because albumin can bind drugs, it can impact how well they are distributed and how effective they are in treating arthritis [56]. Terpinen-4-ol showed the highest docking score against this protein and interacted with Phe402, Val432, Cys437, and Leu452 residue binding sites by hydrophobic bonds and Asn390 via a conventional hydrogen bond of the active site whereas the control binds with this residue which confirmed to predict the antiarthritic activity of the plant extract. Terpinen-4-ol exhibited antiarthritic activity in both in vivo and in vitro analysis in previous experiments [106].

For predicting the antidiabetic activity of the selected compounds, they were docked against human DPP-4 receptor [107]. DPP-4 receptor is vital for glucose homeostasis. It is required for incretin breakdown as incretins allow the body to produce more insulin when it desired and its inhibitors act by boosting incretin levels, which elevate insulin secretion, impede glucagon release, lower blood sugar levels, and slow stomach emptying [108]. Di-n-octyl phthalate showed the highest docking score against human DPP-4 receptor and interacted with Ser209 active site residue via conventional hydrogen bond and Phe357, Arg358, Tyr631, Tyr662, Tyr666 by hydrophobic bonds and Glu205 and Glu206 by carbon–hydrogen bond, whereas the control did not bind with Ser209 and Tyr631 residues of the active site making it a potential compound for exerting antidiabetic effects. Furthermore, to predict the antihyperlipidemic activity of the selected compounds, they were docked against human squalene synthase. Two molecules of farnesyl diphosphate (FPP) are reductively dimerized by squalene synthase to produce squalene, an important precursor to cholesterol. There are two separate phases to the reaction, and both of them feature the generation of carbo-cationic reaction intermediates; this makes the reaction unique among enzymes that use FPP. Due to the fact that FPP is situated at the terminal branch point in the isoprenoid biosynthesis pathway, the conversion of FPP to squalene by means of the action of squalene synthase constitutes the initial committed step in the synthesis of cholesterol [109]. As a result, it is an appealing target for therapeutic intervention. In this study, the compound 8,11,14-docosatrienoic acid methyl ester showed the highest docking score against the target protein and interacted with Phe54, Tyr73, Leu76, Ala176, Leu183, Leu211, Phe288, and Pro292 residues through hydrophobic bonds, whereas the control did not bind with Leu76 and Ala176 residue of the active site making it a strong competitor to predict the antihyperlipidemic activity. As this extract possesses liver enzyme protective activity, there is a higher possibility of displaying hepatoprotective activity for this extract. Therefore, in silico hepatoprotective activity was performed to predict this activity among the identified compounds. For hepatoprotective activity, all the phytochemicals were docked against the TNF-alpha converting enzyme [59]. By cleaving and secreting a number of cell surface proteins, including TNF-alpha, TNF-alpha converting enzyme (TACE) helps regulate inflammatory reactions. Some disorders, especially liver ailments, are linked to dysregulation of the inflammatory cytokine TNF-alpha. There is evidence that TNF-alpha contributes to the development of inflammation and tissue damage in the setting of liver disease. One enzyme that helps activate inflammatory processes is TACE, which is responsible for the release of soluble TNF-alpha. An increase in TNF-alpha levels is common in diseases such as viral hepatitis, alcoholic liver disease (ALD), and non-alcoholic fatty liver disease (NAFLD). TACE activity may play a role in regulating these levels. Drugs have the ability to alleviate liver inflammation by reducing TNF-alpha release via inhibiting TACE [110]. Phenol, 2-methoxy-4-(2-propenyl)-, acetate showed the highest docking score against TNF-alpha converting enzyme and interacted with Ser441 and Gly442 active site residues by a strong conventional hydrogen bond and Leu401, His405, and Ala439 by hydrophobic bonds, whereas the control did not bind with Ser441 and Gly442 residues making it a strong competitor for predicting the hepatoprotective activity. Hence, all the experimental results were supported by one or more compounds identified in the MEMI peel.

The limitation of the study was that the extract was given to the diabetic mice for 7 days. To observe the more precise effects of the extract on glucose, lipid profile, and liver enzyme level in the blood, it is crucial to perform the experiment for a longer period. In addition, the histopathological study is also important to observe the effects of the extract on the heart, kidney, and liver along with biochemical parameter analysis. Other antioxidant methods, such as ABTS radical, hydrogen peroxide, and superoxide anion scavenging assays, are also important to conclude prominent antioxidant activity. These experiments could not be carried out due to inevitable limitations. However, it is our initial effort to perform the research on an untapped cultivar of *M. indica* for its potential pharmacological benefits. As this study focuses on providing a wide array of pharmacological evaluations, the most common methods were utilized. We believe that this study provides new insights for the researchers to develop a novel medication.

## 5. Conclusions

These findings highlight the pharmacological potential of MEMI peel as an antioxidant, antiarthritic, antimicrobial, antidiabetic, and diabetes-related complications. Molecular docking simulations further elucidated the potential mechanisms of action of MEMI peel constituents. Compounds such as di-n-octyl phthalate, terpinen-4-ol, 8,11,14-docosatrienoic acid methyl ester, and phenol 2-methoxy-4-(2-propenyl)-acetate showed strong binding affinity to target proteins associated with antioxidant, antibacterial, antiarthritic, antidiabetic, antihyperlipidemic, and liver enzyme protective activities. However, more in-depth comprehensive studies must be conducted on the MEMI peel of unexplored cultivars to fully elucidate its therapeutic mechanisms and optimize its clinical applications.

## Figures and Tables

**Table 1:** Phytochemicals identified in MEMI peel by GC-MS analysis.

Peak no.	Name of compound	Retention time	Area	Conc. (%)	m/z	Signal/noise	Molecular formula	Chemical class
1	Benzaldehyde dimethyl acetal	5.755	44,075	1.70	121	27.03	C_9_H_12_O_2_	Benzyl ether
2	Terpinen-4-ol	7.180	18,311	0.70	71	11.42	C_10_H_18_O	Terpenoid
3	Azulene	7.295	83,693	3.22	128	42.78	C_10_H_8_	Polycyclic aromatic
4	Estragole	7.500	42,946	1.65	148	33.79	C_10_H_12_O	Phenylpropanoid
5	9-Methoxybicyclo[6.1.0]nona-2,4,6-triene	10.080	56,707	2.18	115	34.24	C_10_H_12_O	Hydrocarbon
6	Phenol, 2-methoxy-4-(2-propenyl)-, acetate	10.565	21,925	0.84	164	17.32	C_12_H_14_O_3_	Phenolics benzoate ester
7	3-Tetradecene, (Z)-	11.285	25,865	0.99	55	3.94	C_14_H_28_	Hydrocarbon
8	Benzenemethanol, alpha-methyl-alpha-propyl-	11.435	103,691	3.99	115	34.77	C_11_H_16_O	Phenolics
9	Caryophyllene	11.910	13,628	0.52	93	10.81	C_15_H_24_	Sesquiterpene
10	10-Methylnonadecane	13.215	35,866	1.38	71	9.71	C_20_H_42_	Hydrocarbon
11	Phenol, 3,5-bis(1,1-dimethylethyl)-	13.705	547,483	21.06	191	312.65	C_14_H_22_O	Phenolics
12	Methyl tetradecanoate	20.305	84,416	3.25	74	39.91	C_15_H_30_O	Fatty acid ester
13	Docosanoic acid, ethyl ester	21.975	20,696	0.80	88	13.19	C_24_H_48_O_2_	Fatty acid ester
14	Hexadecen-1-ol, trans-9-	23.800	91,426	3.52	55	13.92	C_16_H_32_O	Fatty alcohol
15	Tetradecanoic acid, propyl ester	23.960	27,135	1.04	61	14.93	C_17_H_34_O_2_	Fatty acid ester
16	9-Octadecenoic acid (Z)-, methyl ester	24.215	32,008	1.23	55	5.98	C_19_H_36_O_2_	Fatty acid ester
17	Hexadecanoic acid, methyl ester	24.635	630,075	24.24	74	187.43	C_17_H_34_O_2_	Fatty acid ester
18	Octadecanoic acid, ethyl ester	25.830	93,164	3.58	88	45.11	C_20_H_40_O_2_	Fatty acid ester
19	Hexadecanoic acid, propyl ester	27.370	122,881	4.73	61	58.38	C_19_H_38_O_2_	Fatty acid ester
20	6-Octadecenoic acid, methyl ester, (Z)-	27.515	84,192	3.24	55	10.32	C_19_H_36_O_2_	Fatty acid ester
21	(Z)-Methyl hexadec-11-enoate	27.600	16,359	0.63	55	2.39	C_17_H_32_O_2_	Fatty acid ester
22	Hexadecanoic acid, 2-methylpropyl ester	28.180	65,184	2.51	57	7.63	C_20_H_40_O_2_	Fatty acid ester
23	7-Tetradecenal, (Z)-	28.500	17,123	0.66	55	2.40	C_14_H_26_O	Fatty aldehyde
24	Hexadecanoic acid, 1,1-dimethylethyl ester	28.785	35,178	1.35	56	8.98	C_20_H_40_O_2_	Fatty acid ester
25	8,11,14-Docosatrienoic acid, methyl ester	29.845	17,570	0.68	55	2.65	C_23_H_40_O_2_	Fatty acid ester
26	Tetradecane, 2,6,10-trimethyl-	32.840	23,369	0.90	57	4.50	C_17_H_36_	Hydrocarbon
27	Di-n-octyl phthalate	33.220	128,088	4.93	149	43.90	C_24_H_38_O_4_	Ester derivatives
28	9-Octadecenamide, (Z)-	36.020	116,524	4.48	59	13.48	C_18_H_35_NO	Fatty amide

**Table 2:** Antioxidant activity of MEMI peel (IC_50_ value of MEMI peel and the standard).

Sample	IC_50_ (μg/mL)
MEMI peel	4.43 ± 0.68
BHT (standard)	6.53 ± 0.53

**Table 3:** Antibacterial Activity of MEMI peel.

Bacterial strain	Diameter of zone of inhibition (mm)	
	MEMI peel (500 μg/disc)	Kanamycin disc (30 μg/disc)
Gram positive		
*Staphylococcus aureus*	15.33 ± 1.32	25.33 ± 1.12
*Bacillus cereus*	20.67 ± 1.52	21.33 ± 1.76
Gram negative		
*Escherichia coli*	13.33 ± 1.26	15.67 ± 0.76
*Pseudomonas aeruginosa*	15.67 ± 1.86	23.33 ± 1.02

*Note:* Assay was performed in triplicate (*n* = 3), and the results were expressed as mean ± SD.

**Table 4:** Molecular docking binding affinity of the selected compounds against each target proteins.

PubChem CID	Compound names	Docking score (kcal/mol)					
		1DGH	4URM	4OR0	2OQV	1EZF	2FV5
62375	Benzaldehyde dimethyl acetal	−6.0	−5.0	−6.4	−5.6	−5.2	−5.9
11230	Terpinen-4-ol	−6.1	−5.8	−7.6	−5.7	−5.5	−7.7
9231	Azulene	−6.4	−6.0	−6.5	−6.3	−6.5	−6.7
8815	Estragole	−6.6	−5.5	−6.2	−5.0	−5.7	−6.3
5370526	9-methoxybicyclo[6.1.0]nona-2,4,6-triene	−6.9	−5.5	−6.9	−5.1	−5.8	−6.3
7136	Phenol, 2-methoxy-4-(2-propenyl)-,acetate	−6.9	−5.9	−6.5	−5.5	−6.6	−7.8
5362709	3-tetradecene, (Z)-	−5.6	−5.0	−5.9	−4.0	−6.1	−5.9
138214	Benzenemethanol, alpha-methyl-alpha-propyl-	−6.3	−5.6	−6.6	−6.4	−6.2	−7.0
5281515	Caryophyllene	−7.1	−6.5	−7.0	−6.0	−7.5	−5.9
530070	10-Methylnonadecane	−6.9	−4.9	−5.4	−4.4	−6.4	−5.8
70825	Phenol, 3,5-bis(1,1-dimethylethyl)-	−6.9	−5.7	−7.2	−6.1	−7.1	−7.4
31284	Methyl tetradecanoate	−6.4	−5.1	−6.1	−4.1	−6.0	−6.0
22199	Docosanoic acid, ethyl ester	−5.3	−5.2	−5.9	−4.0	−6.3	−5.8
5283282	Hexadecen-1-ol, trans-9-	−5.2	−5.6	−5.5	−4.5	−6.1	−5.9
84338	Tetradecanoic acid, propyl ester	−6.2	−5.3	−6.3	−4.4	−6.0	−6.2
5364509	9-octadecenoic acid (Z)-, methyl ester	−6.5	−4.8	−6.1	−4.9	−6.2	−6.0
8181	Hexadecanoic acid, methyl ester	−5.5	−4.3	−6.7	−4.7	−5.9	−5.3
8122	Octadecanoic acid, ethyl ester	−6.9	−4.5	−6.6	−4.6	−5.9	−5.4
75232	Hexadecanoic acid, propyl ester	−5.7	−4.8	−4.6	−4.6	−6.1	−6.0
5362717	6-octadecenoic acid, methyl ester, (Z)-	−5.9	−5.2	−6.3	−4.7	−6.4	−6.3
11277200	(Z)-methyl hexadec-11-enoate	−5.3	−5.3	−6.7	−4.8	−6.2	−5.9
66967	Hexadecanoic acid, 2-methylpropyl ester	−7.0	−6.5	−6.8	−6.3	−7.5	−6.5
5364468	7-tetradecenal, (Z)-	−6.1	−4.8	−5.6	−4.5	−6.0	−6.2
544739	Hexadecanoic acid, 1,1-dimethylethyl ester	−7.3	−4.7	−6.3	−5.1	−6.0	−6.3
5364473	8,11,14-docosatrienoic acid, methyl ester	−7.1	−5.7	−6.4	−5.0	−7.6	−6.2
85785	Tetradecane, 2,6,10-trimethyl-	−6.8	−4.7	−7.5	−4.5	−6.4	−6.3
8346	Di-n-octyl phthalate	−7.5	−5.9	−6.8	−6.4	−6.7	−7.1
5283387	9-octadecenamide, (Z)-	−7.2	−4.8	−6.0	−4.9	−6.4	−6.3
—	Control (cocrystallized ligand)	−7.8	−7.9	−9.4	−9.9	−11.6	−11.1

**Table 5:** Molecular docking interaction analysis of the top five compounds with human erythrocyte catalase (PDB ID: 1DGH) for antioxidant activity.

Compounds	Docking score (kcal/mol)	Conventional hydrogen bond interactions	Hydrophobic bond interactions	Salt bridge/carbon–hydrogen bonds	Electrostatic (attractive charge/Pi-cation)
Di-n-octyl phthalate	−7.5	His305	Pro151, Phe198, Arg203, Val302, Pro304, Ala445, Phe446, Val450	—	—
Hexadecanoic acid, 1,1-dimethylethyl ester	−7.3	—	Pro129, Phe198, Leu199, Arg203, Ala445, Phe446, Val450	—	—
8,11,14-docosatrienoic acid, methyl ester	−7.2	Asn148, Ser217	Arg72, Val74, Arg112, Ala133, Phe153, Phe161, Phe334, Met350, Arg354, Ala357, Tyr358, His362	Val146	—
9-octadecenamide, (Z)-	−7.1	His305	Pro151, His194, Phe198, Arg203, Tyr215, Val302, Ala445, Val450	—	—
Hexadecanoic acid, 2-methylpropyl ester	−7.0	—	Ala8, Ile262, Arg263, Phe266, Tyr325	—	—
Control (cocrystallized ligand)	−7.8	His194, Ser201, Arg203, Asn213, Lys237, Trp303, His305, Gln442	Pro151, Phe198, Val302, Phe446, Val450	Arg203, His235, His305	Arg203, His235, Lys237

**Table 6:** Molecular docking interaction analysis of the top five compounds with *Staphylococcus aureus* Gyrase B enzyme (PDB ID: 4URM) for antibacterial activity.

Compounds	Docking score (kcal/mol)	Conventional hydrogen bond interactions	Hydrophobic bond interactions	Salt bridge/halogen/carbon–hydrogen bonds	Electrostatic (attractive charge/Pi-cation/Pi-anion)
Di-n-octyl phthalate	−6.5	Glu58	Ile86, Pro87, Ile102	Asn54	Arg84
Caryophyllene	−6.4	—	Ile86, Pro87	—	—
Hexadecanoic acid, 2-methylpropyl ester	−6.1	Gln91	Ile86, Pro87, Val101	—	—
Azulene	−6.0	—	Tyr141, Val174	—	—
Phenol, 2-methoxy-4-(2-propenyl)-, acetate	−5.9	—	Ile51, Ile86, Ile175	Thr173	—
Control (cocrystallized ligand)	−7.9	Glu58, Gln91, Lys93, Gly109, Lys111	Ile86, Pro87, Ala98, Ile102	Ala108	—

**Table 7:** Molecular docking interaction analysis of the top five compounds with bovine serum albumin (PDB ID: 4OR0) for antiarthritic activity.

Compounds	Docking score (kcal/mol)	Conventional hydrogen bond interactions	Hydrophobic bond interactions	Salt bridge/halogen/carbon–hydrogen bonds	Electrostatic (attractive charge/Pi-cation/Pi-anion)
Terpinen-4-ol	−7.6	Asn390	Phe402, Val432, Cys437, Leu452	—	—
Tetradecane, 2,6,10-trimethyl-	−7.5	—	Phe501, Phe506, Phe508, Lys524, Ala527, Leu528, Leu531, His534, Met547, Phe550, Val575	—	—
Phenol, 3,5-bis(1,1-dimethylethyl)-	−7.2	—	Leu115, Tyr160, Arg185, Leu189	—	—
Caryophyllene	−7.0	—	Pro117, Leu122	—	—
9-methoxybicyclo[6.1.0]nona-2,4,6-triene	−6.9	—	Leu429, Val432, Leu452	—	—
Control (cocrystallized ligand)	−9.4	Ser488	Cys391, Leu406, Leu429, Val432, Cys436, Cys437, Leu452	Val432, Thr448	Arg409

**Table 8:** Molecular docking interaction analysis of the top five compounds with human DPP4 receptor (PDB ID: 2OQV) for antidiabetic activity.

Compounds	Docking score (kcal/mol)	Conventional hydrogen bond interactions	Hydrophobic bond interactions	Salt bridge/halogen/carbon–hydrogen bonds	Electrostatic (attractive charge/Pi-cation/Pi-anion)
Di-n-octyl phthalate	−6.4	Ser209	Arg356, Phe357, Arg358, Tyr631, Val656, Trp659, Tyr662, Tyr666	Glu205, Glu206,	
Benzene methanol, alpha-methyl-alpha-propyl-	−6.4	Arg125, Asn710	Ser630, Tyr631, Tyr662, Tyr666	—	—
Azulene	−6.3	—	Ser630, Tyr631, Val656, Tyr662, Tyr666	—	—
Hexadecanoic acid, 2-methylpropyl ester	−6.3	—	Arg356, Phe357, Arg358	—	—
Phenol, 3,5-bis(1,1-dimethylethyl)-	−6.1	Glu205, Arg669	Phe357	—	—
Control (cocrystallized ligand)	−9.9	Glu205, Glu206, Arg358, Tyr547, Tyr662, Tyr666	Phe357, Arg358, Tyr662, Tyr666	His740	—

**Table 9:** Molecular docking interaction analysis of the top five compounds with human squalene synthase (PDB ID: 1EZF) for antihyperlipidemic activity.

Compounds	Docking score (kcal/mol)	Conventional hydrogen bond interactions	Hydrophobic bond interactions	Salt bridge/halogen/carbon–hydrogen bonds	Electrostatic (attractive charge/Pi-cation/Pi-anion)
8,11,14-docosatrienoic acid, methyl ester	−7.6	Asn215	Phe54, Tyr73, Leu76, Ala176, Val179, Leu183, Met207, Leu211, Phe288, Cys289, Pro292	—	—
Hexadecanoic acid, 2-methylpropyl ester	−7.5	—	Phe54, Tyr73, Leu76, Ala176, Val179, Leu183, Phe288, Pro292	—	—
Caryophyllene	−7.5	—	Val179	—	—
Phenol, 3,5-bis(1,1-dimethylethyl)-	−7.1	—	Phe54, Ala176, Val179, Leu211	—	—
Di-n-octyl phthalate	−6.7	—	Phe54, Val69, Phe72, Leu76, Ala176, Val179, Leu183, Met207, Leu211, Pro292	—	Asp80
Control (cocrystallized ligand)	−11.6	Arg52, Ser53, Phe54	Val69, Phe72, Tyr73, Val179, Leu183, Leu211, Phe288, Pro292	—	Cys289

**Table 10:** Molecular docking interaction analysis of the top five compounds with TNF-alpha converting enzyme (PDB ID: 2FV5) for hepatoprotective activity.

Compounds	Docking score (kcal/mol)	Conventional hydrogen bond interactions	Hydrophobic bond interactions	Salt bridge/halogen/carbon–hydrogen bonds	Electrostatic (attractive charge/Pi-cation/Pi-anion)
Phenol, 2-methoxy-4-(2-propenyl)-,acetate	−7.8	Ser441, Gly442	Leu401, His405, Ala439	Ser441	—
Terpinen-4-ol	−7.7	Val434	Leu401, His405, Ala439	—	—
Phenol, 3,5-bis(1,1-dimethylethyl)-	−7.4	—	Leu401, Ala439	—	—
Benzene methanol, alpha-methyl-alpha-propyl-	−7.0	—	Leu348, Leu401, Val402, His405, Ala439	—	—
Di-n-octyl phthalate	−6.4	Ser441	Leu348, Ile394, Leu401, Val402, His405, Ala439	Val440	—
Control (cocrystallized ligand)	−11.1	Leu348, Gly349, Glu406, His409, His415	Leu401, Ala439, Val440	—	His405

## Data Availability

The data used to support the findings will be available from the corresponding author upon request.

## Nomenclature

DPPH: 2,2-diphenyl-1-picrylhydrazyl
TC: Total cholesterol
TG: Triglycerides
LDL: Low-density lipoprotein
HDL: High-density lipoprotein
AST: Aspartate aminotransferase
ALT: Alanine aminotransferase
DPP-4: Dipeptidyl peptidase-4
GC–MS: Gas chromatography–mass spectrometry
ROS: Reactive oxygen species
STZ: Streptozotocin
BSA: Bovine serum albumin
HAS: Human serum albumin
FPP: Farnesyl diphosphate
TNF-alpha: Tumor necrosis factor-alpha
TACE: Tumor necrosis factor-alpha converting enzyme

## Supporting Information

Table S1: Phytochemical analysis of MEMI peel; Table S2: Effect of MEMI peel on acute oral toxicity test in Swiss albino mice; Figure S1: 2D interactions of (A) hexadecanoic acid, 1,1-dimethylethyl ester; (B) 8,11,14-docosatrienoic acid methyl ester; (C) 9-octadecenamide, (Z)-; and (D) hexadecanoic acid, 2-methylpropyl ester with human erythrocyte catalase for antioxidant activity; Figure S2: 2D interactions of (A) caryophyllene; (B) hexadecanoic acid, 2-methylpropyl ester; (C) azulene; and (D phenol, 2-methoxy-4-(2-propenyl)-, acetate with *Staphylococcus aureus* Gyrase B enzyme for antibacterial activity; Figure S3: 2D interactions of (A) tetradecane, 2,6,10-trimethyl-; (B) phenol, 3,5-bis(1,1-dimethylethyl)-; (C) caryophyllene; and (D) 9-methoxybicyclo[6.1.0]nona-2,4,6-triene with BSA for antiarthritic activity; Figure S4: 2D interactions of (A) benzenemethanol, alpha-methyl-alpha-propyl-; (B) azulene; (C) hexadecanoic acid, 2-methylpropyl ester; and (D) phenol, 3,5-bis(1,1-dimethylethyl)- with human DPP4 receptor for antidiabetic activity; Figure S5: 2D interactions of (A) hexadecanoic acid, 2-methylpropyl ester; (B) caryophyllene; (C) phenol, 3,5-bis(1,1-dimethylethyl)-; and (D) di-n-octyl phthalate with human squalene synthase for antihyperlipidemic activity; Figure S6: 2D interactions of (A) terpinen-4-ol; (B) phenol, 3,5-bis(1,1-dimethylethyl)-; (C) benzenemethanol, alpha-methyl-alpha-propyl-; and (D) di-n-octyl phthalate with TNF-alpha converting enzyme for hepatoprotective activity.
