# Supplementary material for: Unveiling Pharmacological Promise of Mangifera indica (Haribhanga) Peel Extract: Exploring an Untapped Cultivar Through Biochemical and Computational Approaches
Source: Scientifica (Cairo). 2025 Mar 10;2025:6516268. doi: 10.1155/sci5/6516268 (PMC11986926; doi:10.1155/sci5/6516268)
Supplement: Supporting Information — Additional supporting information can be found online in the Supporting Information section. [file 6516268.f1.pdf]

Supplementary Materials

Supplementary Tables

Supplementary Table S1: Phytochemical analysis of MEMI peel

Sl. No.	Phytochemicals	Results
1.	Tannins	+
2.	Terpenoids	+
3.	Phenols	+
4.	Flavonoids	+
5.	Alkaloids	-
6.	Saponins	+
7.	Glycosides	+

Note: (+) = Present (-) = Absent

Supplementary Table S2: Effect of MEMI peel on acute oral toxicity test in Swiss albino mice

SI. No.	Response	Before Treatment	After Treatment						
			25 mg/kg	50 mg/kg	100 mg/kg	250 mg/kg	500 mg/kg	1000 mg/kg	2000 mg/kg
1	Alertness	Normal	Normal	Normal	Normal	Normal	Normal	Normal	Normal
2	Grooming	Absent	Absent	Absent	Absent	Absent	Absent	Absent	Absent
3	Restlessness	Absent	Absent	Absent	Absent	Absent	Absent	Absent	Absent
4	Touch response	Normal	Normal	Normal	Normal	Normal	Normal	Normal	Normal
5	Pain response	Normal	Normal	Normal	Normal	Normal	Normal	Normal	Normal
6	Tremors	Absent	Absent	Absent	Absent	Absent	Absent	Absent	Absent
7	Convulsion	Absent	Absent	Absent	Absent	Absent	Absent	Absent	Absent
8	Righting reflex	Normal	Normal	Normal	Normal	Normal	Normal	Normal	Normal
9	Gripping	Normal	Normal	Normal	Normal	Normal	Normal	Normal	Normal
10	Pinna reflex	Present	Present	Present	Present	Present	Present	Present	Present
11	Corneal reflex	Present	Present	Present	Present	Present	Present	Present	Present
12	Urination	Normal	Normal	Normal	Normal	Normal	Normal	Normal	Normal
13	Writhing	Absent	Absent	Absent	Absent	Absent	Absent	Absent	Absent
14	Salivation	Normal	Normal	Normal	Normal	Normal	Normal	Normal	Normal
15	Pupils	Normal	Normal	Normal	Normal	Normal	Normal	Normal	Normal
16	Lacrimation	Normal	Normal	Normal	Normal	Normal	Normal	Normal	Normal
17	Skin color	Normal	Normal	Normal	Normal	Normal	Normal	Normal	Normal
18	Food intake	Normal	Normal	Normal	Normal	Normal	Normal	Normal	Normal
19	Water intake	Normal	Normal	Normal	Normal	Normal	Normal	Normal	Normal
20	Mortality	Not applicable	Nil	Nil	Nil	Nil	Nil	Nil	Nil

Supplementary Figures

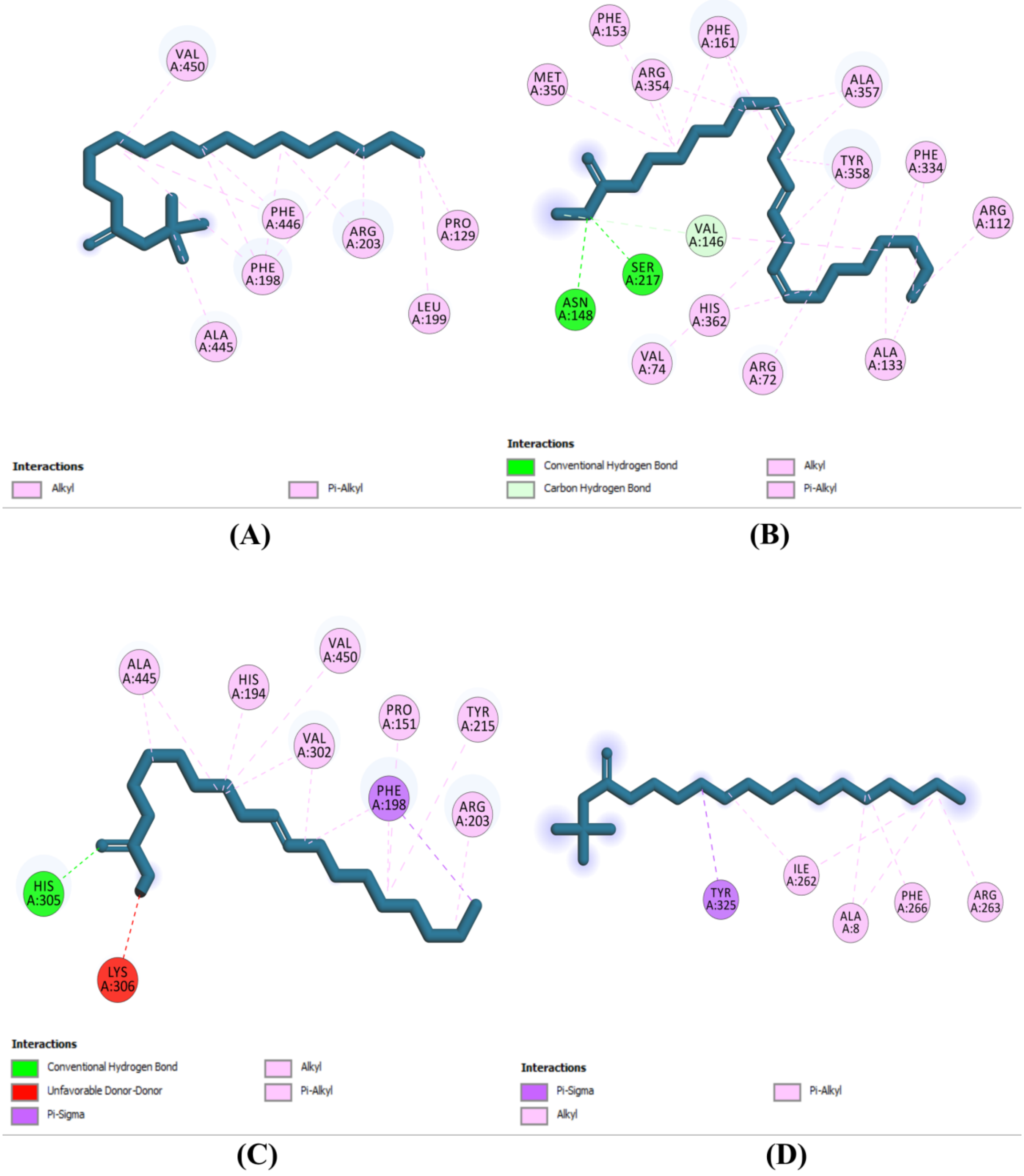

Supplementary Figure S1: 2D interactions of (A) hexadecanoic acid, 1,1-dimethylethyl ester, (B) 8,11,14-docosatrienoic acid, methyl ester, (C) 9-octadecenamide, (Z)- and (D) hexadecanoic acid, 2-methylpropyl ester with human erythrocyte catalase for antioxidant activity

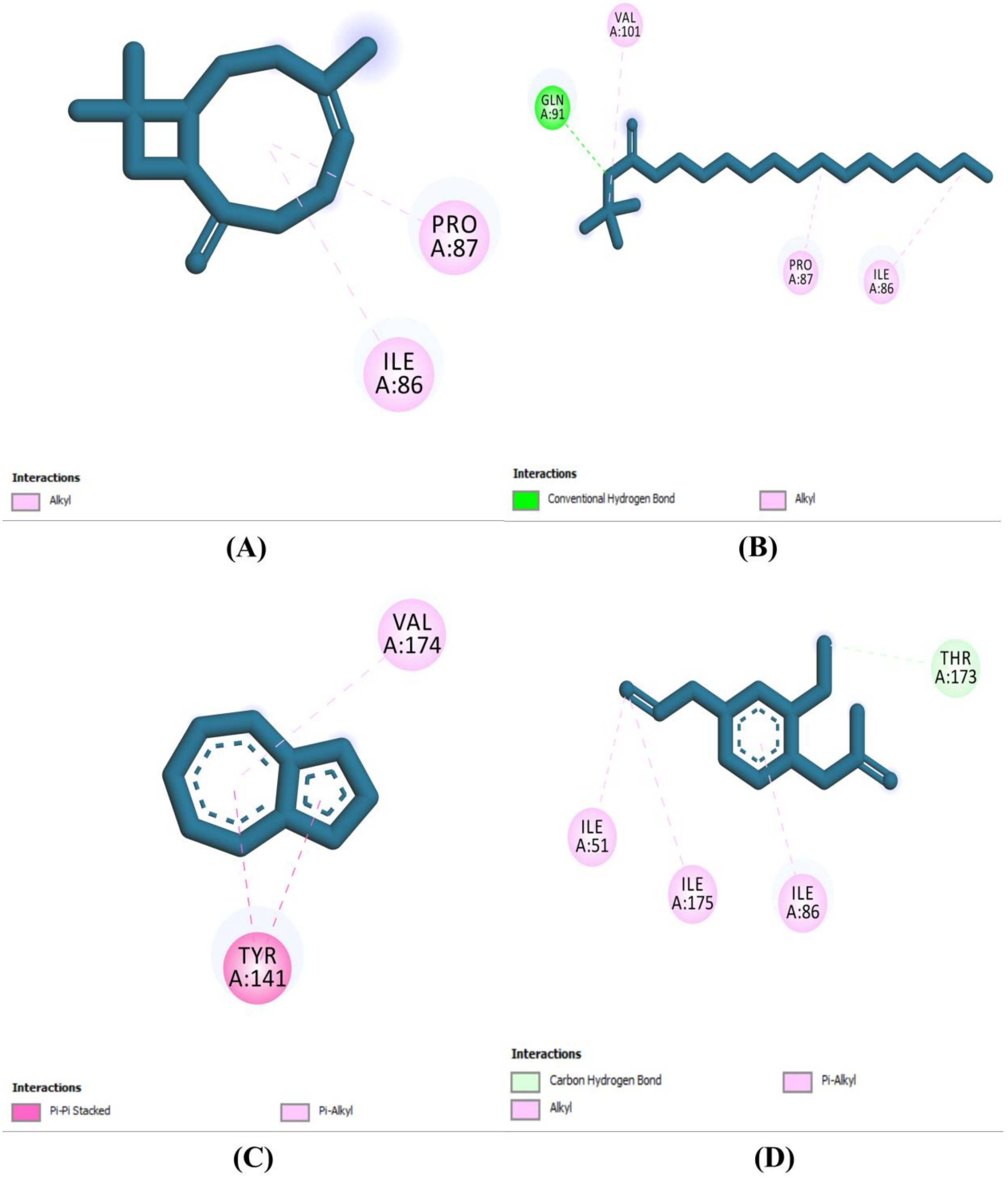

Supplementary Figure S2: 2D interactions of (A) caryophyllene, (B) hexadecanoic acid, 2-methylpropyl ester, (C) azulene and (D phenol, 2-methoxy-4-(2-propenyl)-,acetate with Staphylococcus Aureus Gyrase B enzyme for antibacterial activity

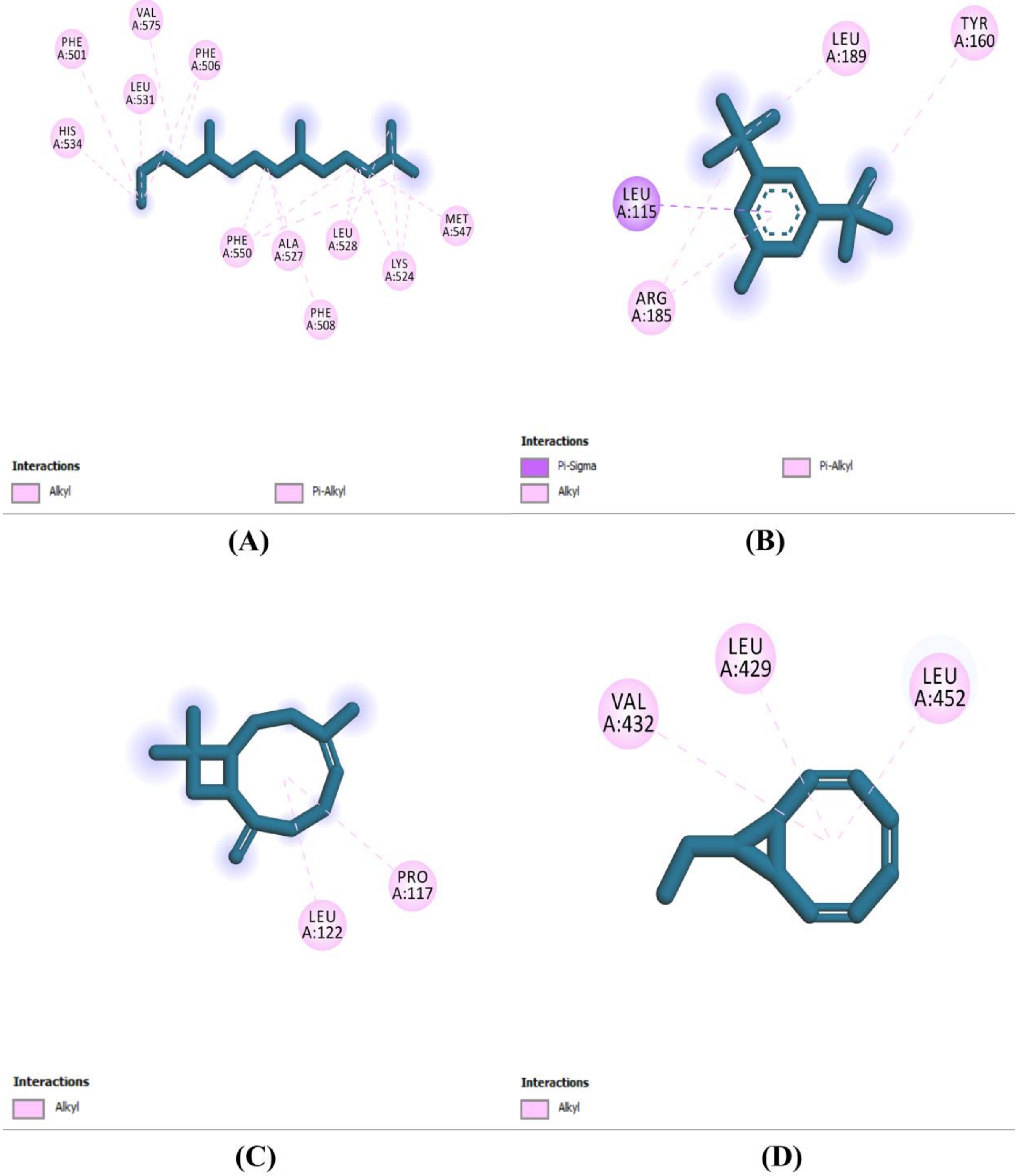

Supplementary Figure S3: 2D interactions of (A) tetradecane, 2,6,10-trimethyl-, (B) phenol, 3,5-bis(1,1-dimethylethyl)-, (C) caryophyllene, and (D) 9-methoxybicyclo[6.1.0]nona-2,4,6-triene with bovine serum albumin for antiarthritic activity

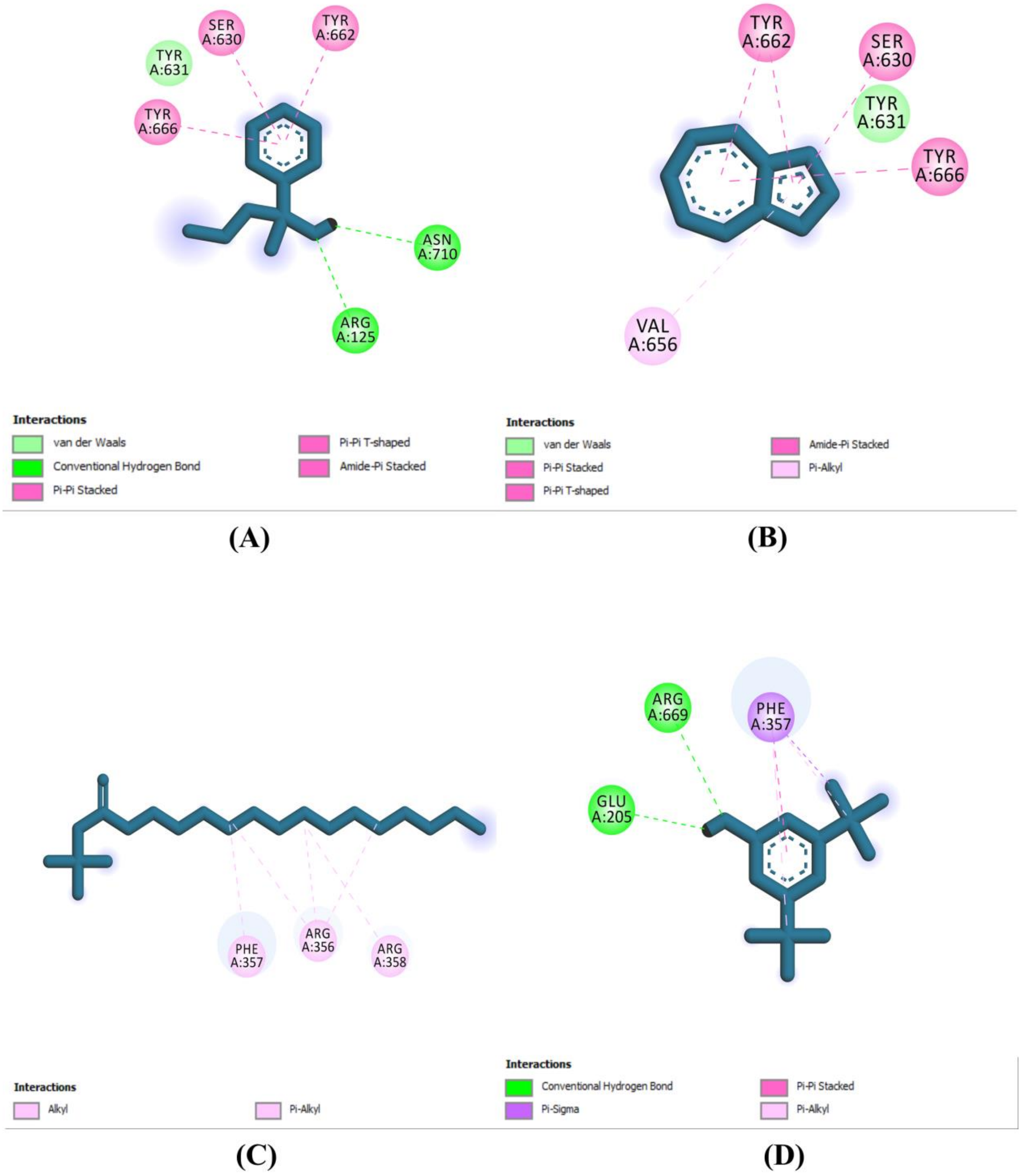

Supplementary Figure S4: 2D interactions of (A) benzenemethanol, .alpha.-methyl-.alpha.-propyl-, (B) azulene, (C) hexadecanoic acid, 2-methylpropyl ester, and (D) phenol, 3,5-bis(1,1-dimethylethyl)- with human DPP4 receptor for antidiabetic activity

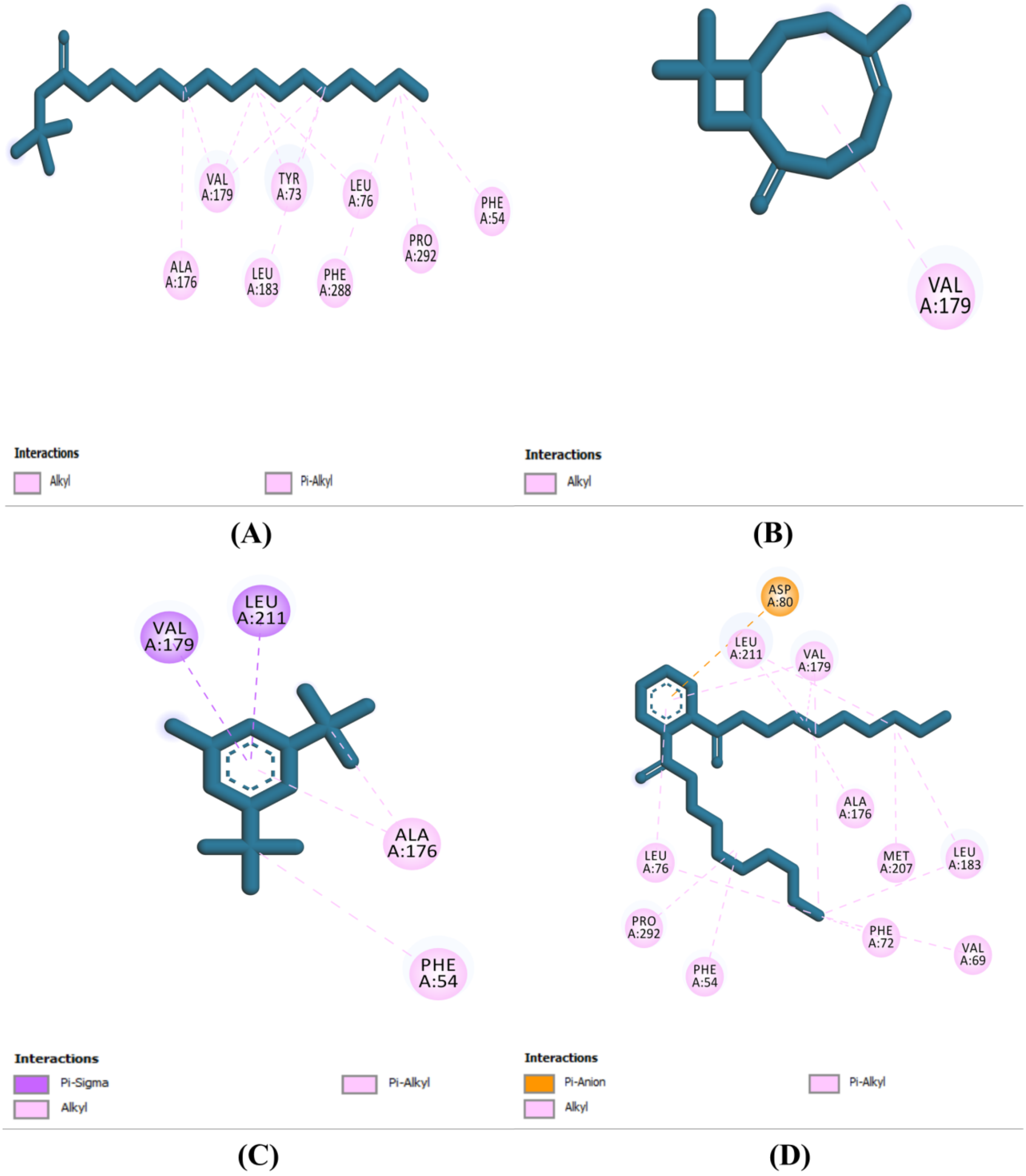

Supplementary Figure S5: 2D interactions of (A) hexadecanoic acid, 2-methylpropyl ester, (B) caryophyllene, (C) phenol, 3,5-bis(1,1-dimethylethyl)-, and (D) di-n-octyl phthalate with human squalene synthase for antihyperlipidemic activity.

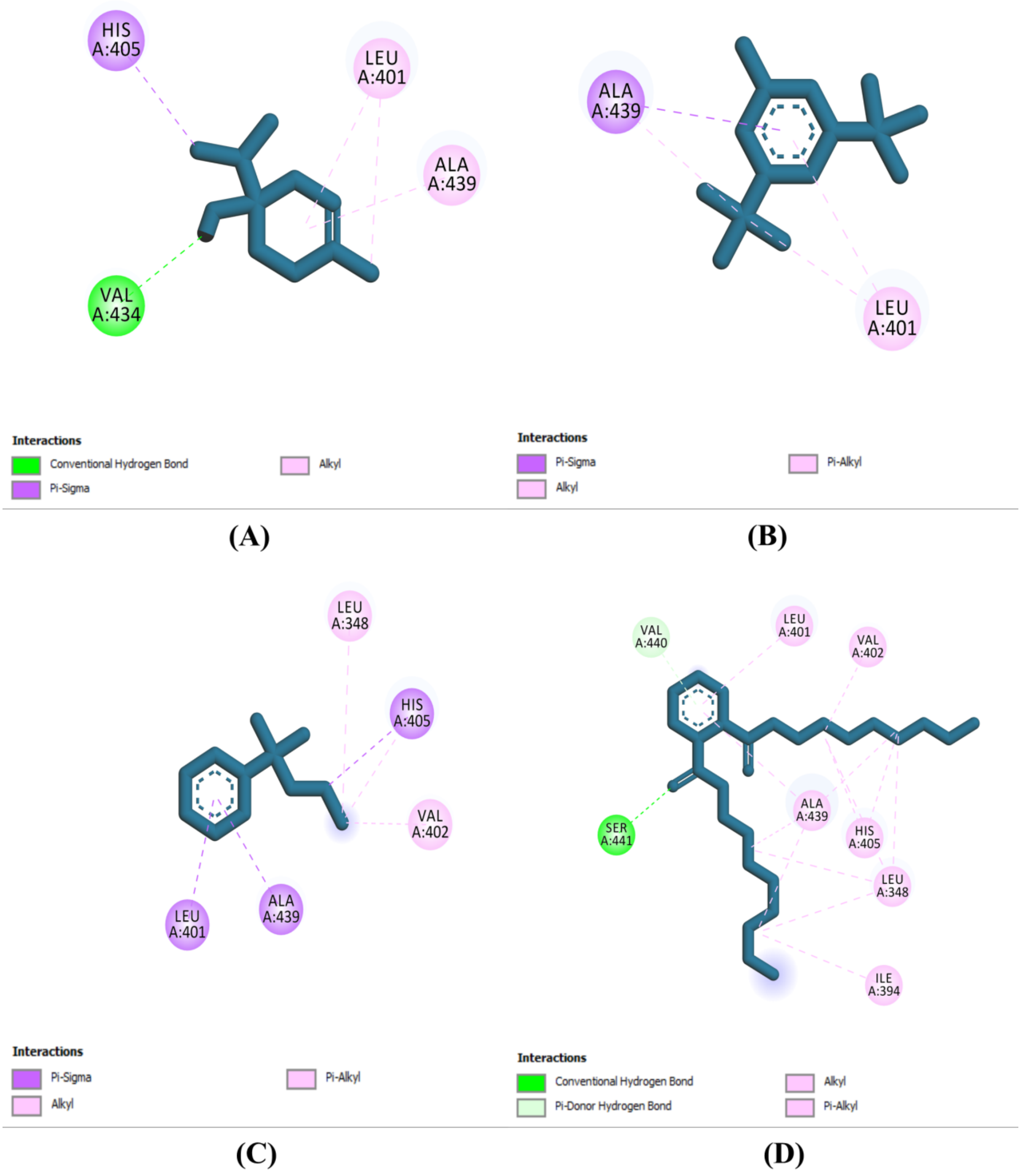

Supplementary Figure S6: 2D interactions of (A) terpinen-4-ol, (B) phenol, 3,5-bis(1,1-dimethylethyl)-, (C) benzenemethanol, .alpha.-methyl-.alpha.-propyl-, and (D) di-n-octyl phthalate with TNF-alpha converting enzyme for hepatoprotective activity.
